# Single cell tuning of Myc expression by antigen receptor signal strength and interleukin-2 in T lymphocytes

**DOI:** 10.15252/embj.201490252

**Published:** 2015-07-01

**Authors:** Gavin C Preston, Linda V Sinclair, Aneesa Kaskar, Jens L Hukelmann, Maria N Navarro, Isabel Ferrero, H Robson MacDonald, Victoria H Cowling, Doreen A Cantrell

**Affiliations:** 1 Department of Cell Signalling & Immunology, College of Life Sciences, University of DundeeDundee, UK; 2 Centre for Gene Regulation and Expression, College of Life Sciences, University of DundeeDundee, UK; 3 Instituto Investigación Sanitaria/Hospital Universitario de la Princesa, Universidad Autónoma de MadridMadrid, Spain; 4 Ludwig Center for Cancer Research of the University of LausanneEpalinges, Switzerland

**Keywords:** cytokine signals, metabolism, Myc, T lymphocytes, TCR signals

## Abstract

Myc controls the metabolic reprogramming that supports effector T cell differentiation. The expression of Myc is regulated by the T cell antigen receptor (TCR) and pro-inflammatory cytokines such as interleukin-2 (IL-2). We now show that the TCR is a digital switch for Myc mRNA and protein expression that allows the strength of the antigen stimulus to determine the frequency of T cells that express Myc. IL-2 signalling strength also directs Myc expression but in an analogue process that fine-tunes Myc quantity in individual cells via post-transcriptional control of Myc protein. Fine-tuning Myc matters and is possible as Myc protein has a very short half-life in T cells due to its constant phosphorylation by glycogen synthase kinase 3 (GSK3) and subsequent proteasomal degradation. We show that Myc only accumulates in T cells exhibiting high levels of amino acid uptake allowing T cells to match Myc expression to biosynthetic demands. The combination of digital and analogue processes allows tight control of Myc expression at the population and single cell level during immune responses.

See also: **C Chou & T Egawa** (August 2015)

## Introduction

Immune-activated T lymphocytes undergo rapid clonal expansion and differentiation into effector subpopulations. Activated T cells also strikingly increase nutrient uptake and lipid and protein biosynthesis. Naïve T lymphocytes thus have low rates of amino acid and glucose uptake and use oxidative phosphorylation to efficiently metabolise glucose to generate ATP. In contrast, effector T cells upregulate amino acid and glucose uptake and switch to metabolising glucose through glycolysis (Greiner *et al*, 1994; Fox *et al*, 2005; Jacobs *et al*, 2008); they also metabolise glutamine to pyruvate and lactate via glutaminolysis (Brand *et al*, 1984; Newsholme *et al*, 1985). These changes in T cell metabolism are necessary to support the production of the cytokines and cytolytic effector molecules that are essential for T cell immune responses (Pollizzi & Powell, 2014).

One key controller of metabolic reprogramming in T cells is Myc (myelocytomatosis oncogene, c-Myc). In the absence of Myc, mature T cells cannot respond to antigen receptor engagement to increase the expression of glucose and glutamine transporters and fail to initiate glycolysis or glutamine catabolism (Wang *et al*, 2011a). Myc loss also has a global impact on the T cell transcriptome. Myc may thus have multiple transcriptional targets or act to amplify expression of active genes in T cells (Nie *et al*, 2012). The importance of Myc in T lymphocytes is also exemplified by its role in T cell development in the thymus (Dose *et al*, 2006, 2009; Mycko *et al*, 2009; Jiang *et al*, 2010) and by its key role in T cell malignancies. For example, elevated Myc expression is observed in most cases of T cell acute lymphoblastic leukaemia (T-ALL), either as a consequence of a chromosomal translocation event (t(8;14)(q24;q11)) that places Myc under the control of the TCR alpha chain promoter (Erikson *et al*, 1986; Charrin, 1996) or more commonly as a consequence of Notch mutations that lead to transcriptional activation of Myc (Weng *et al*, 2004, 2006; Palomero *et al*, 2006).

It was recognised many years ago that upregulation of Myc mRNA accompanied T cell activation (Reed *et al*, 1987; Kelly & Siebenlist, 1988; Wang *et al*, 2011a). More recently, the Immunological Genome Project has mapped the transcriptional profiles of multiple T cell populations and reported high levels of Myc mRNA in immune-activated effector T cells (Best *et al*, 2013). However, the expression of Myc mRNA and protein can be quite discordant because of post-transcriptional control of Myc protein (Vervoorts *et al*, 2006; Junttila & Westermarck, 2008; Thomas & Tansey, 2011; Ehninger *et al*, 2014). For example, stabilisation of Myc protein by a T58A mutation has been described in Burkitt’s lymphoma (Yano *et al*, 1993; Gregory & Hann, 2000) and inactivating mutations of the E3 ubiquitin ligase Fbw7 that target Myc for proteasomal degradation are frequently found in T-ALL (Welcker *et al*, 2004a; O’Neil *et al*, 2007).

The relevance of understanding the control of Myc protein levels in T cells was highlighted recently: immune-activated T cells lacking the system L amino acid transporter Slc7a5 upregulate Myc mRNA but fail to express Myc protein and hence have global metabolic defects (Sinclair *et al*, 2013). In this context, many experiments have analysed Myc mRNA in T cells activated pharmacologically with phorbol esters, calcium ionophores and plant lectins (Kelly *et al*, 1983; Reed *et al*, 1987, 1988; Kelly & Siebenlist, 1988), but there is almost no detailed analysis of Myc protein in T cells responding to physiological stimuli. Accordingly, the present study has explored how engagement of the T cell antigen receptor (TCR) with peptide/major histocompatibility complexes (p/MHC) controls Myc expression. We have also examined how a key pro-inflammatory cytokine interleukin-2 (IL-2) impacts on Myc expression. We show that the TCR is a digital switch that couples the strength of the antigen stimulus to the frequency of cells within a population that express Myc. However, TCR triggering alone cannot sustain expression of Myc and post-transcriptional control of Myc protein by IL-2, a γc cytokine that triggers Janus-associated kinase (JAK)-dependent signalling pathways, fine-tunes Myc levels in individual T cells. We show that Myc is constantly phosphorylated by the serine/threonine kinase GSK3 in activated T cells and targeted for proteasomal degradation. Myc protein thus only accumulates in T cells with high levels of protein synthesis. This study also affords the insight that the cellular concentration of Myc matters in the context of T cell metabolism.

## Results

### TCR signal strength determines Myc protein expression in a digital manner

**Figure 1:**
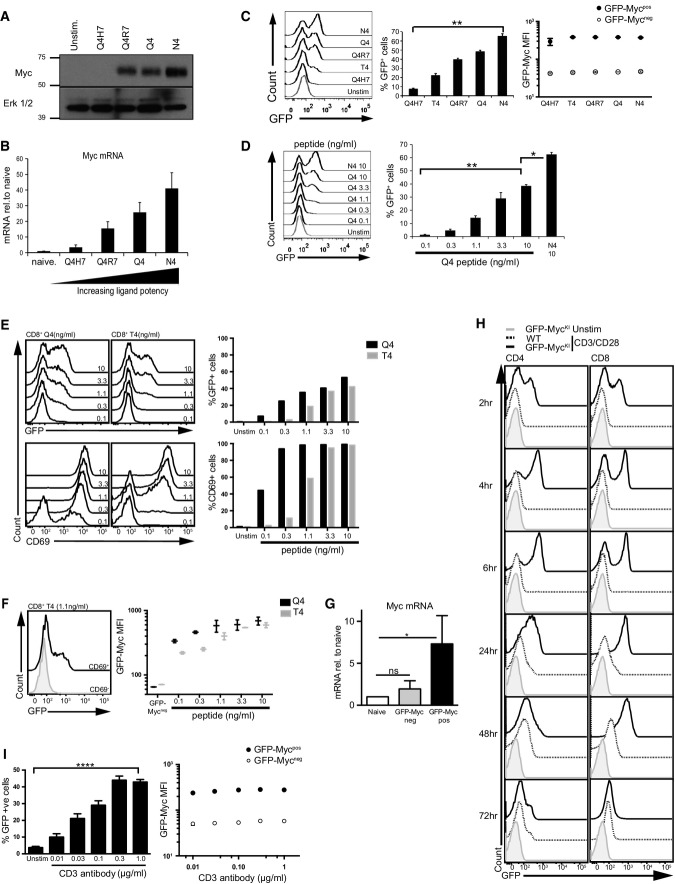
T cell receptor signalling drives digital expression of Myc A, B CD8^+^ T cells were purified from OT1 lymph node cells that had been stimulated for 4 h with 10 ng/ml of peptides SIIQFEHL (Q4H7), SIIQFERL (Q4R7), SIIQFEKL (Q4) or SIINFEKL (N4) or were left unstimulated. (A) Western blot data show Myc and ERK1/2 protein expression, representative of 3 biological replicates. (B) qPCR data show Myc mRNA expression relative to unstimulated naïve control (*n *= 3, mean ± SEM).
C–F Flow cytometry data from lymph node cells of OT1 GFP-Myc^KI^ mice stimulated through the TCR with peptide or left unstimulated. Data are from at least 3 biological replicates. (C) GFP-Myc expression in CD8^+^ T cells stimulated for 2 h with 10 ng/ml peptides Q4H7, T4, Q4R7, Q4 or N4 (left panel). The middle panel shows the percentage GFP-Myc^pos^ cells (mean ± SEM, ANOVA was used to determine statistical significance, **P <* *0.01). The right panel shows the MFIs of the GFP^pos^ and GFP^neg^ populations (mean ± SEM). (D) GFP-Myc expression in CD8^+^ T cells stimulated for 2 h with varying concentrations of Q4 (ng/ml) or N4 (10 ng/ml) (left panel). The right panel shows the percentage GFP-Myc^pos^ cells (mean ± SEM, ANOVA was used to determine statistical significance, *P <* *0.05, ** *P *<* *0.01). (E) GFP-Myc (top panel) and CD69 (bottom panel) expression on CD8^+^ T cells stimulated for 20 h with varying concentrations of Q4 and T4 peptides, indicated on the histograms. The right panels show the percentage of GFP-Myc^pos^ or CD69^+^ populations. (F) GFP-Myc expression in CD69^+^ or CD69^−^ CD8^+^ T cells stimulated with 1.1 ng/ml of T4 peptide for 20 h (left panel). The right panel shows the GFP-Myc MFI of the CD69^+^ populations of cells treated as in (E) (mean ± SEM). 

The expression of Myc in T cells is controlled by the TCR (Wang *et al*, 2011a). A crucial question is how TCR signalling strength controls the pool size of T cells that differentiate to effector cells. In this respect, although Myc is required for T cell differentiation, the impact of antigen signalling strength on Myc expression has not been explored. To explore the sensitivity of Myc induction to different strengths of TCR ligand, we used the OT1 TCR transgenic model where there are well-characterised peptide ligands with different affinities for the TCR (Daniels *et al*, 2006). These permit exploration of how the affinity of peptide-major histocompatibility complexes (p/MHC), the physiological ligand for TCR complexes, dictates Myc expression. In these experiments, we activated OT1 T cells with TCR agonists of varying affinity: SIIQFEHL (Q4H7: *K*_d_ 51 ± 9.1 nM), SIITFEKL (T4: *K*_d_ of 55 ± 10.1 nM), SIIQFERL (Q4R7: *K*_d_ 48 ± 9.5 nM), SIIQFEKL (Q4: *K*_d_ 29 ± 7.2 nM) and SIINFEKL (N4: *K*_d_ 3.7 ± 0.7 nM) (Daniels *et al*, 2006). Western blot analysis of OT1 T cells shows that the level of Myc protein within a T cell population is determined by the strength of the TCR ligand (Fig1A). Cellular levels of Myc mRNA are also determined by TCR ligand strength (Fig1B), revealing that one fundamental way TCR agonists control Myc expression in T cells is by controlling the expression of Myc mRNA.

G GFP-Myc^neg^ and GFP-Myc^pos^ CD8^+^ T cells were purified from OT1 GFP-Myc^KI^ lymph node cells that had been activated for 2 h with N4 peptide (1 ng/ml) or had been left unstimulated. Graph shows the expression of Myc mRNA measured by qPCR relative to naïve unstimulated CD8^+^ T cells (*P <* *0.05, ANOVA was used to determine statistical significance). Data show mean+SEM of 3 biological replicates.

H, I Flow cytometry data from lymph node cells of WT or GFP-Myc^KI^ mice stimulated using CD3 and CD28 antibodies or left unstimulated. Data are from at least 3 biological replicates. (H) GFP-Myc expression over time in CD4^+^ or CD8^+^ T cells following stimulation with CD3 (1 μg/ml) and CD28 (3 μg/ml) antibodies. (I) The left panel shows the percentage GFP-Myc^pos^ CD8^+^ T cells stimulated with varying concentrations of CD3 (μg/ml as on graph) and CD28 (3 μg/ml) antibodies for 4 h. The right panel shows the MFIs of the GFP-Myc^pos^ and GFP-Myc^neg^ populations (mean ± SEM, ****P < 0.001, ANOVA was used to determine statistical significance). Source data are available online for this figure.

Changes in Myc levels in response to TCR ligand strength could reflect that ligand affinity governs the frequency of T cells within a population that express Myc. Alternatively, TCR ligand strength may dictate the amount of Myc expressed by each individual activated T cell. To distinguish these possibilities, we needed to directly visualise Myc protein expression at the single cell level. Accordingly, we used a mouse model in which a fusion protein of Myc and enhanced green fluorescent protein (GFP-Myc) is expressed from the endogenous Myc locus (GFP-Myc^KI^) (Huang *et al*, 2008). We then used OT1 GFP-Myc^KI^ T cells to assess whether the strength of the TCR ligand influenced the frequency of T cells expressing Myc within a population. Fig1C shows the pattern of Myc expression in OT1 T cells activated with TCR agonists of varying affinity for the TCR. GFP-Myc is not expressed in naïve CD8^+^ T cells but is rapidly induced following exposure to TCR peptide agonists (2 h). Following TCR engagement, two distinct cell populations were observed: a population with high GFP-Myc expression and a population with no detectable Myc expression (Fig1C, left panel). It was striking that the frequency of T cells that express Myc is determined by the strength of the TCR ligand (Fig1C, middle panel). This on/off pattern of Myc expression suggested a bimodal/digital response pattern of Myc regulation. Consistent with a digital response, the mean fluorescence intensity (MFI) of Myc expression in the GFP-Myc^pos^ populations is not significantly different for the T4, Q4R7, Q4 or N4 peptides (Fig1C, right panel). Hence, increasing the affinity of the TCR ligand does not increase the amount of Myc in individual responding cells. We also examined Myc expression following naïve OT1 T cell activation with increasing concentrations of the p/MHC ligand Q4. Fig1D shows that the concentration of TCR ligand dictates the frequency of T cells that express Myc.

The bimodal, on/off pattern of Myc expression was also maintained during a sustained (20 h) T cell response to p/MHC ligands (Fig1E). Here we compared the TCR ligand dose–response for induction of Myc and the activation marker CD69. Fig1E (bottom panel) shows that the upregulation of CD69 is more sensitive to the TCR ligand dose than Myc induction (top panel). The expression of CD69 marks T cells that have responded to antigen, and in this respect, it was of note that Myc expression was restricted to the CD69-expressing T cells and was bimodal in the CD69^pos^ TCR-activated lymphocytes (Fig1F, left panel). We did however note that at this sustained time point, the level of p/MHC ligand not only directed the frequency of T cells that initiated Myc expression but also influenced the level of Myc per cell (Fig1F, right panel).

One important question was whether the frequency of cells that expressed Myc protein in activated T cells was explained by differences in the frequency of T cells that express Myc mRNA. To answer this question, OT1 GFP-Myc^KI^ T cells were activated with the N4 TCR agonist for 2 h before GFP-Myc^pos^ and GFP-Myc^neg^ T cells were purified by FACS and Myc mRNA was quantified. Fig1G shows that N4-stimulated GFP-Myc^pos^ OT1 T cells express high levels of Myc mRNA compared with GFP-Myc^neg^ OT1 T cells. This result is consistent with the hypothesis that the TCR controls the frequency of cells that express Myc because the TCR controls the frequency of cells that express Myc mRNA.

We next examined whether digital Myc expression is common to both CD4^+^ and CD8^+^ T cells. Fig1H shows that GFP-Myc is not expressed in naïve lymph node-derived CD4^+^ and CD8^+^ T cells but is rapidly induced in both populations following polyclonal T cell activation with CD3 and CD28 antibodies with a clear digital response pattern of Myc regulation. In these experiments, we used saturating levels of CD3 antibodies, and within the first few hours of the activation response, there was an increase in the percentage of T cells that expressed Myc but no increase in the maximal amount of Myc expressed. Under these potent activation conditions, all T cells expressed Myc at the 24-h time point. This response was sustained over a 72-h period, but it was notable that the level of Myc per cell had decreased at the 48- and 72-h time points (Fig1H).

When T cells were activated polyclonally with different concentrations of CD3 antibody, then the frequency of Myc-expressing cells directly correlated with the amount of CD3 antibody used (Fig1I), and the digital nature of the response was preserved. In these experiments, the level of the costimulatory antibody CD28 was constant, indicating that it is the level of TCR engagement that determined Myc expression. The digital nature of Myc induction in response to TCR engagement is thus a common feature of the TCR response to both physiological and polyclonal ligands and occurs in both CD4^+^ and CD8^+^ T cells.

### IL-2/JAK signalling controls Myc expression in TCR-activated T cells

**Figure 2:**
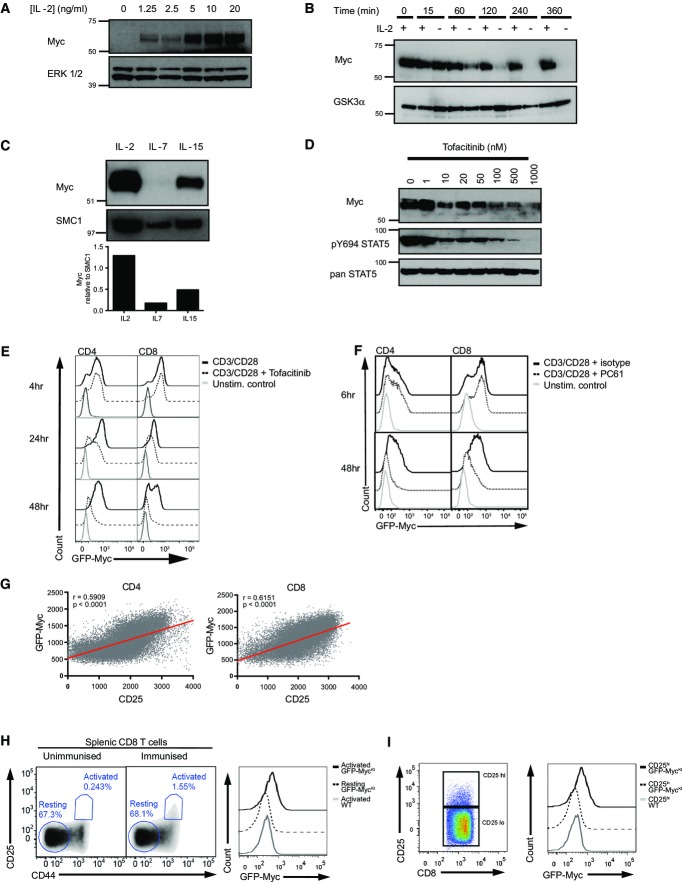
γc cytokine signalling supports Myc expression in an analogue fashion A–D CD8^+^ CTL were generated from splenocytes as described in Materials and Methods and maintained in IL-2 (20 ng/ml). Data are representative of at least 3 experiments. (A) Western blot data show Myc and ERK1/2 protein expression in CTL switched into the indicated concentration of IL-2 for 2 h. (B) Myc and GSK3α protein expression in CTL maintained in IL-2 or deprived of IL-2 for the times indicated. (C) Myc and SMC1 expression in CTL maintained in IL-2 or switched into IL-15 (20 ng/ml) or IL-7 (5 ng/ml) for 18 h. Histogram shows densitometry analysis of Myc expression relative to SMC1 expression. (D) Myc, pY694STAT5 and panSTAT5 protein expression in CTL after 2-h treatment with the JAK inhibitor tofacitinib at the indicated concentrations.

The differentiation of antigen-activated CD8^+^ T cells is regulated by γc cytokines such as interleukin-2 (IL-2). Antigen-primed T cells cultured in IL-2 thus differentiate to effector cytotoxic T cells (CTL) (Kalia *et al*, 2010; Pipkin *et al*, 2010). It has also been described that IL-2 controls the expression of Myc (Reed *et al*, 1988; Lord *et al*, 2000). In this context, we observed that antigen receptor-triggered T cells maintained in culture in the presence of receptor-saturating levels of IL-2 expressed high levels of Myc (Fig2A). Moreover, it was striking that sustained IL-2 signalling is required to maintain the high expression of Myc protein in CTL, with Myc levels decreasing within 1 h of withdrawal from IL-2 (Fig2B). We also examined the effect of two other γc cytokines, IL-7 and IL-15, on the expression of Myc in CTL. In this context, Myc expression is essential *in vivo* for signalling by IL-15 (Bianchi *et al*, 2006; Dose *et al*, 2006, 2009; Mycko *et al*, 2009; Jiang *et al*, 2010). Fig2C shows that IL-2, IL-15 and IL-7 are different in their ability to maintain Myc protein expression in effector CTL. IL-2 has the strongest effect, IL-15 is much less potent at maintaining Myc expression, and there are only very low levels of Myc in effector CTL maintained in IL-7.

E Lymph node cells from GFP-Myc^KI^ mice were stimulated using CD3 (1 μg/ml) and CD28 (3 μg/ml) antibodies in the presence or absence of JAK inhibitor tofacitinib (100 nM). Flow cytometry data show GFP-Myc expression in CD4^+^ (left) or CD8^+^ (right) T cells at the indicated times. Data are representative of at least 3 experiments.

F Lymph node cells from GFP-Myc^KI^ mice were stimulated using CD3 (1 μg/ml) and CD28 (3 μg/ml) antibodies in the presence of IL-2 receptor-blocking antibody PC61 (2 μg/ml) or isotype control for 6 or 48 h. Flow cytometry data show GFP-Myc expression in CD4^+^ (left) or CD8^+^ (right) T cells. Data are representative of 3 biological replicates.

G T cells from lymph nodes of GFP-Myc^KI^ mice were activated with CD3 antibody (60 ng/ml) for 48 h. The plots show the fluorescence intensity of GFP-Myc and CD25 expression from flow cytometry data modelled by linear regression in CD4^+^ cells (left panel) and CD8^+^ cells (right panel). Data are representative of 3 experiments.

H, I GFP-Myc^KI^ KI and WT mice were immunised with attenuated *Listeria monocytogenes,* and after 24 h, spleens were harvested for analysis. Data are from 2 independent experiments with 2 control and 3 test animals in each experiment. (H) Flow cytometry data showing the gating of activated (CD25^pos^ and CD44^pos^) and resting (CD25^neg^ and CD44^neg^) CD8^+^ T cells from control (left panel) and immunised (middle panel) mice. The right panel shows GFP-Myc expression of activated and resting CD8^+^ T cells with the corresponding MFI values. (I) Flow cytometry data showing the gating of CD25^high^ and CD25^low^ CD8^+^ T cells (left panel) and corresponding GFP-Myc expression in the GFP-Myc^KI^ cells compared to WT cells (right panel). Source data are available online for this figure.

IL-2 and IL-15 signal via a receptor complex that includes the common gamma chain (γc) and a β subunit (CD122). Triggering of this receptor complex activates the tyrosine kinases JAK1 and JAK3. IL-2 is able to sustain a much higher level of signalling in activated T cells than IL-15, even when both cytokines are at the receptor-saturating concentrations (Cornish, 2006). The differential effect of IL-2 and IL-15 on Myc expression suggests that the level of JAK kinase activity might determine the expression of Myc. Recently, inhibitors of JAK kinases have been described, notably tofacitinib (Changelian *et al*, 2003). We could therefore test directly the contribution of JAK signalling to the regulation of Myc expression in IL-2-maintained CTL. Fig2D shows that tofacitinib treatment leads to a rapid loss of Myc protein expression in IL-2-maintained CTL.

Immune-activated T cells produce IL-2 and can have autocrine responses to this cytokine (Feau *et al*, 2011; Sa *et al*, 2013). The ability of IL-2 to control Myc expression in CTL made us question whether autocrine IL-2/JAK signalling had any role in controlling Myc expression in TCR-activated naïve T cells. We therefore examined the effect of the JAK inhibitor tofacitinib on Myc induction in TCR- and CD28-activated T cells. Fig2E shows that there was no effect of tofacitinib on Myc expression in T cells activated with CD3 and CD28 antibodies for 4 h, whereas there was a clear reduction in Myc expression in tofacitinib-treated T cells at the 24- and 48-h time point. It was notable that treatment of activated T cells with the JAK inhibitor did not change the frequency of activated T cells that express Myc but rather reduced the amount of Myc expression per cell. In further experiments, we examined the impact of PC61, a CD25 antibody that blocks IL-2 binding to its high-affinity receptor (Lowenthal *et al*, 1985), on Myc expression in T cells activated polyclonally with CD3 and CD28 antibodies. Fig2F shows that blockade of IL-2 binding to its receptor reduced Myc expression in the longterm-activated T cells. However, the neutralising IL-2 antibody had no impact on the digital TCR-induced Myc response seen at 6 h. Inhibition of autocrine IL-2 did not reduce the frequency of activated T cells that express Myc but could reduce Myc expression level per cell. Hence, IL-2 control of Myc expression is an analogue response unlike the bimodal response pattern for TCR control of Myc expression.

The data above argue that there is no role for autocrine IL-2/JAK signalling in the immediate response to TCR engagement, but during the sustained immune activation response, IL-2 signalling does have an impact on Myc expression. In further experiments to explore this question, we took advantage of the fact that the level of IL-2 signalling in a T cell is determined by the level of CD25 expression (Cantrell & Smith, 1984). Moreover, CD25 expression in T cells is controlled by a positive feedback mediated by JAK activation of the transcription factor STAT5 (Nakajima *et al*, 1997). If Myc expression in effector T cells is determined by the strength of IL-2 signalling, then Myc levels will be highest in cells that express high levels of CD25. Fig2G shows that the level of Myc protein in activated CD4^+^ and CD8^+^ T cells correlates with the expression of CD25. Similarly, when GFP-Myc^KI^ mice are immunised with an attenuated strain of *Listeria monocytogenes* over 24 h, it is possible to identify immune-activated CD25-positive effector T cells (Fig2H, left panels). These activated CD8^+^ T cells express Myc, whereas no Myc expression is detected in non-responding naïve CD8^+^ T cells from the same animal (Fig2H, right panel). Importantly, Myc expression levels in the activated CD8^+^ T cells correlate with the level of CD25 expression (Fig2I). Collectively, these data are consistent with the hypothesis that IL-2 activation of JAK signalling pathways controls cellular levels of Myc in effector T cells.

### Transcriptional and post-transcriptional control of Myc expression in T cells

**Figure 3:**
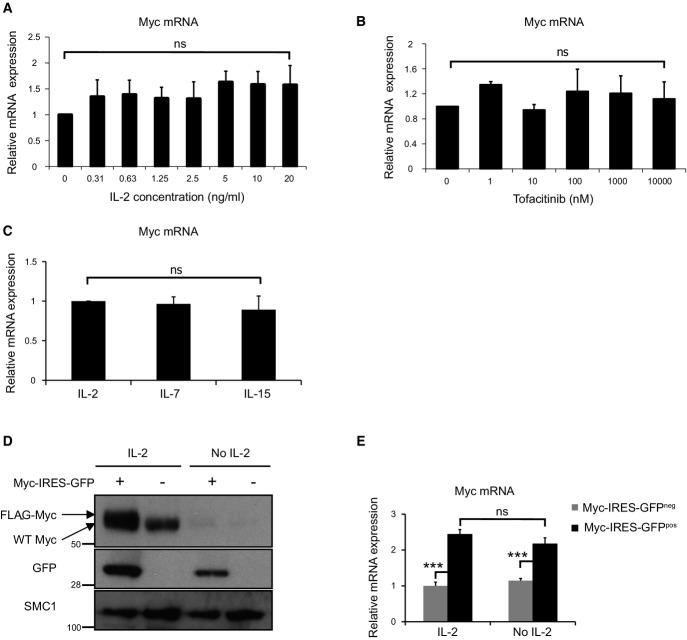
Post-transcriptional regulation of Myc protein expression by γc cytokine signalling A Myc mRNA expression in CTL, generated as described in Materials and Methods, switched into decreasing concentrations of IL-2 for 2 h, shown relative to IL-2-deprived CTL (2 h) (*n* = 3, mean ±* *SEM).

T cell antigen receptor control of Myc expression was explained by TCR control of the frequency of cells that express Myc mRNA. IL-2 regulates an analogue response that controls the amount of Myc expressed by each cell. We therefore assessed whether the analogue IL-2 response reflected the control of Myc mRNA levels. Fig2A shows that although there is a clear IL-2 dose–response for Myc protein expression, there is no equivalent IL-2 dose–response for Myc mRNA in CTL (Fig3A). Similarly, the JAK inhibitor tofacitinib causes CTL to rapidly lose Myc protein but not Myc mRNA (Figs2D and 3B). Moreover, CTL maintained in IL-2, IL-15 or IL-7 have very different levels of Myc protein but express equivalent levels of Myc mRNA (Figs2C and 3C). These data argue that the γc cytokines IL-2 and IL-15 primarily regulate Myc levels via post-transcriptional mechanisms.

B Myc mRNA expression in IL-2-maintained CTL treated with the indicated concentration of tofacitinib for 2 h, shown relative to untreated IL-2-maintained CTL (*n* =* *3, mean ±* *SEM).

C Myc mRNA expression in CTL switched into IL-7 (5 ng/ml) or IL-15 (20 ng/ml) or maintained in IL-2 (20 ng/ml) for 18 h, shown relative to IL-2-maintained CTL (*n* =* *3, mean ±* *SEM).

D, E Myc-IRES-GFP-transduced CD8^+^ cells were sorted by FACS, and the Myc-IRES-GFP^pos^ and Myc-IRES-GFP^neg^ CTL were maintained in IL-2 (20 ng/ml) or deprived of IL-2 for 2 h. Data are representative of 3 experiments. (D) Western blot data show the expression of Myc, GFP and SMC1. (E) qPCR data show Myc mRNA expression relative to IL-2-maintained control (Myc-IRES-GFP^neg^) CTL (mean ±* *SEM, ANOVA used to determine statistical significance of multiple comparisons, ***P <* *0.001). Source data are available online for this figure.

To rigorously test this model further, we transduced activated T cells with a FLAG-Myc^WT^-IRES-GFP retroviral construct (encoding a FLAG-tagged wild-type Myc cDNA and an IRES-controlled GFP cDNA) and then cultured the cells in the presence or absence of IL-2. CTL transduced with the Myc-IRES-GFP vector only overexpress ectopic Myc protein if the cells are cultured with IL-2 (Fig3D), whereas the IRES-controlled GFP (Fig3D) and Myc mRNA (Fig3E) were detected in both the IL-2-maintained and IL-2-deprived T cells. These data show that in the absence of IL-2, CTL cannot sustain the expression of Myc from endogenous or ectopically expressed retroviral Myc transcripts.

### Myc protein has a short half-life in T cells

**Figure 4:**
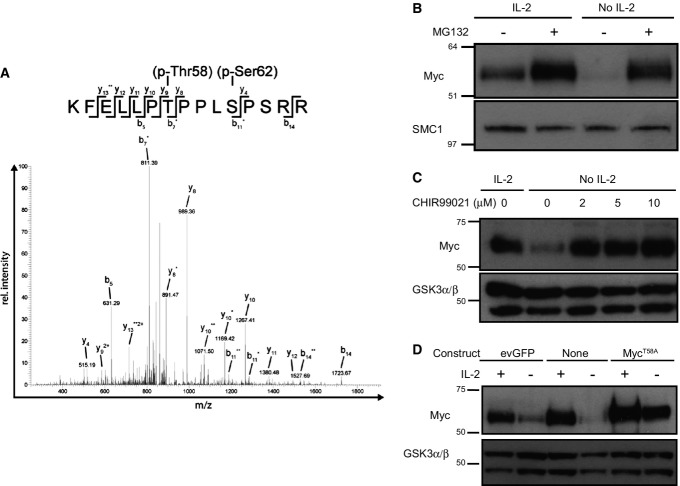
Myc protein expression is post-transcriptionally regulated in response to IL-2 signals by GSK3 and proteasome activity in CTL A Sequence data from phospho-proteomic analysis of IL-2-maintained CTL. Phosphorylated Myc in CTL demonstrated by manual sequencing of acquired multistage activation spectra for the Myc peptide KFELLPTPPLSPSRR (amino acid residues 52–66) with Biemann–Roepstorff nomenclature; asterisks (*y* and *b* ion series) indicate neutral loss of one (*) or two (**) phosphate groups; 2+ indicates double-charged fragment ions. Above: vertical lines indicate a fragmented bond after collision-induced dissociation; horizontal lines indicate the fragment retaining the charge. Data are representative of three experiments.

One striking observation was the rapidity with which Myc protein was lost from CTL following IL-2 withdrawal (Fig2B). These data argue that Myc has a very short half-life in activated T cells. One previously described mechanism to control the expression of Myc is a phosphorylation-dependent pathway that targets Myc for proteasomal degradation (Gregory & Hann, 2000). Phosphorylation of Myc on T58 by glycogen synthase kinase 3β (GSK3β) promotes its interaction with Fbw7, the substrate recognition component of the SCF^Fbw7^ ubiquitin ligase, and directs Myc ubiquitination and proteasomal degradation (Gregory *et al*, 2003; Welcker *et al*, 2004a,b). We have previously used high-resolution mass spectrometry to map the phosphoproteome of IL-2-maintained CTL (Navarro *et al*, 2011). Analysis of this data revealed that Myc is phosphorylated on Ser62 and Thr58 in activated T cells (Fig4A). In this respect, it has been described that the phosphorylation of Ser62 primes Myc for subsequent Thr58 phosphorylation by GSK3β (Sears *et al*, 1999, 2000). These data argue that Myc is constantly phosphorylated and targeted for degradation in IL-2-maintained CTL. To explore this hypothesis further, we examined the impact of MG132, a well-characterised inhibitor of the 26S proteasome, on the expression of Myc in IL-2-maintained and IL-2-deprived CTL. These data show that MG132 promoted the expression of Myc in IL-2-maintained CTL and could also prevent the loss of Myc expression that normally accompanies IL-2 withdrawal (Fig4B). Moreover, if CTL were treated with the GSK3 inhibitor CHIR99021, they were able to sustain Myc expression following IL-2 withdrawal (Fig4C).

B, C CTL were cultured in the presence or absence of IL-2 and inhibitor as indicated for 2 h. Data are representative of 3 independent experiments. (B) Western blot data show Myc and SMC1 expression in CTL treated with or without proteasome inhibitor MG132 (25 μM). (C) Western blot data show Myc and GSK3α/β expression in CTL treated with or without increasing concentration of the GSK3 inhibitor CHIR99021.

D evGFP- or Myc^T58A^-transduced CD8^+^ CTL were sorted by FACS for GFP expression. evGFP, Myc^T58A^ and non-transduced CTL were then maintained in IL-2 (20 ng/ml) or deprived of IL-2 for 18 h. Western blot data show Myc and GSK3α/β expression. Data information: (C, D) Data are from the same lysates but run on parallel gels. Data are representative of 3 experiments. Source data are available online for this figure.

These data indicate that the short half-life of Myc in T cells is controlled by GSK3β-mediated phosphorylation of Myc Thr58, which then targets Myc for proteasomal degradation. This predicts that a Myc protein with the Thr58 mutated to a non-phosphorylatable residue such as alanine would be expressed in T cells independent of the presence of γc cytokines. To test this hypothesis, we transduced antigen receptor-activated T cells with a retroviral construct encoding Myc^T58A^-IRES-eGFP and then cultured the cells in the presence or absence of IL-2. The data show that Myc^T58A^ expression is sustained in CTL deprived of IL-2 (Fig4D). This is in marked contrast to the inability of IL-2-deprived T cells to sustain the expression of endogenous or ectopically expressed retroviral wild-type Myc (Fig3D).

**Figure 5:**
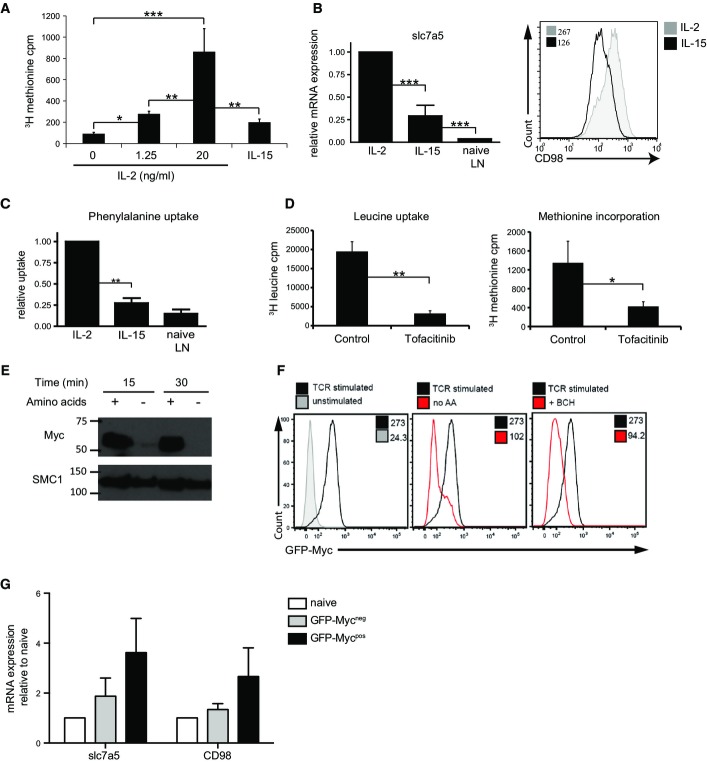
Myc protein expression is dependent on sustained system L amino acid uptake and protein synthesis in CD8^+^ T cells ^3^H methionine incorporation into CTL maintained in IL-15 or IL-2 or switched from IL-2 into varying concentrations of IL-2 (as shown) for 18 h.

The present results demonstrate that GSK3 constantly phosphorylates Myc on Thr58 in T cells and hence constantly targets Myc for degradation. Moreover, it was striking that the treatment of T cells with the proteasome inhibitor MG132 causes Myc to accumulate in IL-2-maintained CTL (Fig4B). This demonstrates that there is a constant high rate of Myc proteolysis by the proteasome in IL-2-maintained T cells. Accordingly, Myc will only be able to accumulate in activated T cells when rates of Myc synthesis exceed the rates of Myc degradation. IL-2 is very potent at inducing amino acid uptake and protein synthesis in CTL (Cornish, 2006; Sinclair *et al*, 2013). High rates of amino acid uptake and consequently high rates of protein synthesis in IL-2-maintained CTL could therefore explain why Myc accumulates in IL-2-maintained CTL and could also explain why IL-15 is unable to sustain high levels of Myc in CTL: IL-15 is much less potent than IL-2 in its ability to promote protein synthesis (Fig5A).

Data show qPCR analysis of slc7a5 mRNA in CTL maintained in IL-2, IL-15 or naïve lymph node cells (left panel). The data are expressed relative to IL-2-maintained CTL. The right panel shows flow cytometry data of CD98 expression on CTL maintained in IL-2 or IL-15, with corresponding MFIs.

^3^H phenylalanine uptake in CTL maintained in IL-2, IL-15 or naïve lymph node cells. The data are expressed relative to IL-2-maintained cells.

^3^H leucine uptake (left panel) and ^3^H methionine incorporation (right panel) measured in IL-2-maintained CTL treated with or without JAK inhibitor tofacitinib (100 nM) for 18 h.

Western blot data show Myc and SMC1 expression in CTL maintained in IL-2 with or without amino acids in culture media for 15 or 30 min.

Lymph node cells from GFP-Myc^KI^ mice were stimulated with CD3 (1 μg/ml) and CD28 (3 μg/ml) for 18 h. Flow cytometry data (left panel) show GFP-Myc expression in CD8^+^ T cells stimulated through the TCR (black) or unstimulated (grey) compared to CD8^+^ T cells stimulated in the absence of amino acids (red, middle panel) and CD8^+^ T cells stimulated in the presence of the system L transporter inhibitor BCH (50 mM) (red, right panel). MFIs are indicated on each panel.

GFP-Myc^neg^ and GFP-Myc^pos^ CD8^+^ T cells were purified from OT1 GFP-Myc^KI^ lymph node cells that had been activated for 2 h with N4 peptide (1 ng/ml). Graph shows expression of slc7a5 and CD98 mRNA measured by qPCR relative to naïve unstimulated CD8^+^ T cells. Data information: (A–E, G) Data shown are from at least 3 experiments (mean ± SEM, *P <* *0.05, **P <* *0.01, ***P <* *0.001, Multiple analyses were performed with ANOVA, comparisons between two groups were performed using Student’s *t*-test.). (F) Data are representative of 3 biological replicates. Source data are available online for this figure.

Recently, we have shown that expression of system L amino acid transporter Slc7a5 by TCR-activated cells is essential for the expression of Myc protein (Sinclair *et al*, 2013). IL-2 can sustain high levels of Slc7a5 expression and CD98, the two subunits of the system L amino acid transporter (Fig5B), and system L-mediated amino acid transport in CTL (Fig5C). In contrast, IL-15 is much less potent at inducing Slc7a5 and CD98 expression (Fig5B) and IL-15-maintained cells exhibit much lower rates of system L amino acid transport (Fig5C). We considered whether the ability of the JAK inhibitor tofacitinib to decrease Myc expression in T cells could be caused by a requirement of JAK activity for system L amino acid uptake and protein synthesis in activated T cells. Fig5D addresses this question and reveals a striking reduction of system L transport activity and very low rates of protein synthesis in CTL treated with tofacitinib.

These results collectively argue that T cells need continual high rates of amino acid uptake to sustain high levels of Myc protein. To test this hypothesis directly, we looked at the impact of amino acid deprivation on Myc expression in activated T cells; indeed, amino acid deprivation caused IL-2-maintained CTL to rapidly lose the expression of Myc (Fig5E). Additionally, blockade of system L transport by the inhibitor 2-amino-2-norbornanecarboxylic acid (BCH) also prevented the upregulation of Myc protein in activated CD8^+^ T cells (Fig5F). Moreover, TCR-stimulated GFP-Myc^pos^ OT1 T cells have high levels of Slc7a5 and CD98 mRNA compared to GFP-Myc^neg^ OT1 T cells (Fig5G). Thus, Myc protein levels in immune-activated T cells are matched to high expression of amino acid transporters and to high rates of amino acid uptake. One consideration in these experiments is that inhibition of amino acid transport will cause loss of mTORC1 activity (Sinclair *et al*, 2013). However, we have shown previously that inhibition of mTORC1 does not prevent Myc expression in TCR- or IL-2-activated CD8^+^ T cells (Finlay *et al*, 2012; Sinclair *et al*, 2013).

### Myc levels are important for activated T cells

**Figure 6:**
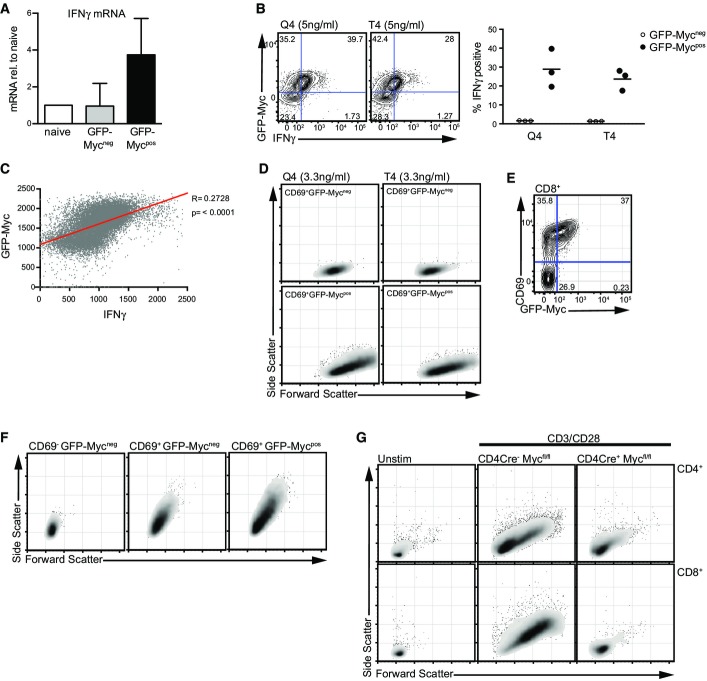
Myc protein expression correlates with effector functions A Lymph node CD8^+^ T cells were activated with N4 peptide (1 ng/ml) for 2 h, and GFP-Myc^neg^ and GFP-Myc^pos^ populations were FACS-sorted. Expression of IFNγ mRNA measured by qPCR relative to naïve unstimulated CD8^+^ T cells is shown. Data show mean + SEM of 3 biological replicates.

The mechanisms whereby TCR signalling strength controls the pool size of T cells that differentiate to effector cells are not fully understood. In the absence of Myc, T cells cannot respond to TCR triggering to proliferate or differentiate to effector cells (Wang *et al*, 2011b). The present data now show that when GFP-Myc^KI^ OT1 T cells are activated with p/MHC ligands, it is the GFP-Myc^pos^ cells that express the effector cytokine interferon gamma (IFNγ) mRNA, whereas GFP-Myc^neg^ cells from the same cultures do not (Fig6A). Moreover, in the sustained responses to p/MHC ligands, it is the GFP-Myc-expressing OT1 T cells that produce high levels of IFNγ and not the GFP-Myc^neg^ T cells (Fig6B). We also observed that the level of IFNγ in an individual cell correlates with the level of expression of GFP-Myc (Fig6C).

B Flow cytometry data show GFP-Myc and IFNγ expression from spleen OT1 GFP-Myc^KI^ CD8^+^ T cells stimulated with 5 ng/ml Q4 (left panel) or T4 (middle panel) peptides for 24 h. Quadrant percentages are shown. The right panel shows the percentage of GFP-Myc^pos^ and GFP-Myc^neg^ CD8^+^ T cells expressing IFNγ protein. Data are from 3 biological replicates.

C The fluorescence intensity of GFP-Myc and IFNγ expression from flow cytometry data modelled by linear regression in OT1 GFP-Myc^KI^ CD8^+^ T cells treated with 5 ng/ml Q4 for 24 h. Data are representative of 3 biological replicates.

D Forward scatter and side scatter plots of CD69^+^ GFP-Myc^neg^ (upper panels) and CD69^+^ GFP-Myc^pos^ (lower panel) OT1 GFP-Myc^KI^ CD8^+^ T cells stimulated with 3.3 ng/ml Q4 or N4 peptides for 24 h. Data are representative of 2 biological replicates

E, F GFP-Myc^KI^ CD8^+^ T cells were stimulated with CD3 antibody (60 ng/ml) for 24 h. Data are representative of 3 biological replicates. (E) Flow cytometry data show CD69 and GFP-Myc expression. (F) Forward and side scatter plots of CD69^−^ GFP-Myc^neg^ (left panel), CD69^+^ GFP-Myc^neg^ (middle panel) and CD69^+^ GFP-Myc^pos^ (right panel) populations.

G CD4Cre^−^ Myc^fl/fl^ and CD4cre^+^ Myc^fl/fl^ lymph node cells were activated with CD3 (1 μg/ml) and CD28 (3 μg/ml) antibodies for 24 h. The data show forward and side scatter plots of CD4^+^ (upper panels) and CD8^+^ (lower panels) T cells, compared to unstimulated CD4Cre^−^ Myc^fl/fl^ cells. Data are representative of at least 3 biological replicates.

T cell antigen receptor engagement triggers a growth response in T cells causing them to increase in cell size (blastogenesis) as judged by flow cytometric analysis of the forward and side scatter properties of activated T cells. We thus used flow cytometry to analyse the forward and side scatter properties of GFP-Myc^KI^ OT1 T cells activated with the p/MHC ligands Q4 and T4. The expression of Myc in the CD69^+^ TCR-activated cells had a bimodal response as described in Fig1. Fig6D shows that it is the GFP-Myc-expressing cells that have undergone blastogenesis. We also analysed blastogenesis of GFP-Myc^KI^ T cell populations activated polyclonally with CD3 and CD28 antibodies. As described above, when T cells are activated polyclonally with different concentrations of CD3 antibody, then the frequency of Myc-expressing cells directly correlated with the dose of TCR ligand used. Fig6E shows GFP-Myc and CD69 expression in CD8^+^ T cells stimulated with a low concentration of the CD3 antibody. A large percentage of the cells responded to TCR ligation to induce CD69 expression, but only a subset of the CD69^+^ cells express GFP-Myc; that is, Myc induction is bimodal in the TCR-activated CD69^+^ T cells. Fig6F shows that CD69^+^ CD8^+^ T cells that do not express Myc have increased cell size in comparison with the CD69^−^ quiescent CD8 T cells. However, the Myc-expressing CD69^+^ CD8^+^ T cells are much larger than the Myc-negative CD69^+^ CD8^+^ T cells. Importantly, T cells with both Myc alleles deleted do not blast in response to TCR and CD28 activation (Fig6G).

**Figure 7:**
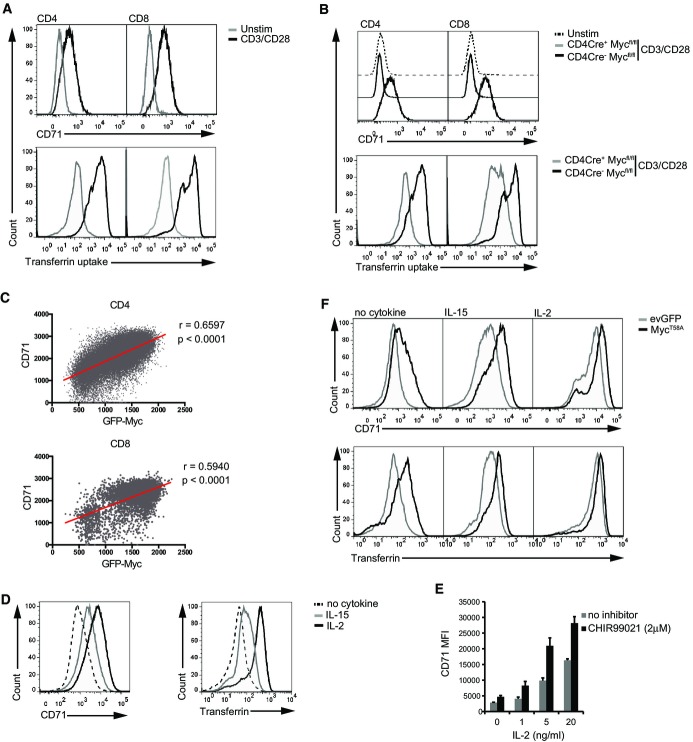
Transferrin receptor (CD71) expression and transferrin uptake are dependent on the level of Myc expression in CD8^+^ T cells A, B Lymph node cells were stimulated using CD3 (1 μg/ml) and CD28 (3 μg/ml) antibodies for 48 h, and unstimulated controls were maintained in IL-7 (5 ng/ml). (A) Histograms show CD71 expression (upper panels) and transferrin uptake (lower panels) in CD4^+^ and CD8^+^ T cells. (B) Histograms show CD71 expression (upper panels) and transferrin uptake (lower panels) in CD4^+^ and CD8^+^ T cells from CD4Cre^+^ Myc^fl/fl^ and CD4Cre^−^ Myc^fl/fl^ control mice.

How important is it that the amount of Myc protein expressed in a single, activated T cell can be fine-tuned? Strategies to address this issue require the ability to quantitatively analyse a Myc-regulated response in single T cells. In this regard, there is strong evidence that Myc regulates transferrin receptor expression (O’Donnell *et al*, 2006) (Holland *et al*, 2012). Moreover, there are good tools for single cell analysis of this pathway that would allow a direct analysis of the importance of Myc protein levels for transferrin uptake. We first addressed whether Myc was indeed important for transferrin receptor expression in T cells. Naïve T cells do not express the transferrin receptor (CD71), but expression of this receptor, and the cellular uptake of transferrin, is strongly induced in antigen receptor-activated T cells (Fig7A). T cells with both Myc alleles deleted cannot upregulate CD71 or transferrin uptake in response to TCR engagement (Fig7B). Is the level of Myc expression per cell important for transferrin receptor expression? To explore this, we first used the GFP-Myc^KI^ mouse model and examined CD71 expression versus GFP-Myc expression in TCR-activated CD4^+^ and CD8^+^ T cells. The data show a clear correlation between expression of CD71 and Myc (Fig7C).

C T cells from lymph nodes of GFP-Myc^KI^ mice were activated with CD3 antibody (60 ng/ml) for 48 h. The plots show the fluorescence intensity of GFP-Myc and CD71 expression from flow cytometry data modelled by linear regression in CD4^+^ cells (upper panel) and CD8^+^ cells (lower panel).

D Flow cytometry data showing CD71 expression and transferrin uptake (bottom panel) in CTL maintained in IL-2 (20 ng/ml) or IL-15 (20 ng/ml) or withdrawn from IL-2 for 18 h (no cytokine).

E CD71 MFI data (mean ± SEM) in CTL treated with decreasing concentrations of IL-2 in the presence or absence of the GSK3 inhibitor CHIR99021 (2 μM) for 18 h.

F evGFP- or Myc^T58A^-transduced CD8^+^ CTL were cultured in IL-2 (20 ng/ml), IL-15 (20 ng/ml) or withdrawn from IL-2 for final 18 h (no cytokine). Histograms show CD71 expression (upper panels) and transferrin uptake (lower panels) as measured by flow cytometry. Data information: (A–D, F) Data are representative of at least 3 biological replicates. (E) Data are quantified from 3 experiments.

There was also a strong correlation between cellular levels of Myc and expression of transferrin receptors in CTL cultured in the cytokines IL-2 and IL-15. Fig7D thus shows that IL-2-maintained CTL express high levels of CD71 and uptake high amounts of transferrin compared to T cells deprived of cytokines, with expression of CD71 and transferrin uptake in activated T cells cultured in IL-15 being intermediate. This correlates with the ability of IL-2 to maintain high levels of Myc protein compared to IL-15 (Fig2C). These data are correlative; hence, to test whether differences in Myc expression levels were causal for differences in CD71 expression, we examined the impact of increasing Myc levels in T cells cultured under these different conditions. In these experiments, we looked at the impact of expressing the Myc^T58A^ mutant on CD71 expression and transferrin uptake because the use of this mutant bypasses the GSK3-mediated pathway that rapidly targets Myc for degradation and allows overexpression of Myc even when cells are cytokine-deprived. In this respect, Fig7E shows that GSK3 inhibition using CHIR99021, which prevents Myc degradation, could increase CD71 expression in IL-2-maintained CTL. There was also a strong effect of Myc^T58A^ expression on CD71 expression and the ability of cells to take up transferrin in cytokine-maintained CTL. Hence, ectopic expression of Myc^T58A^ increased expression of CD71 and transferrin uptake in both IL-15- and even IL-2-cultured CTL (Fig7F). Ectopic expression of Myc^T58A^ was also able to sustain expression of CD71 and transferrin uptake in IL-2-deprived CTL. In addition, increasing Myc levels increased transferrin uptake in IL-15-cultured CTL to the level seen in the control IL-2-maintained cells. Hence, the limiting factor for transferrin receptor expression and transferrin uptake in IL-15-maintained CTL was the level of Myc expression. Moreover, the fact that expression of Myc^T58A^, which increased cellular concentrations of Myc, increases rates of transferrin uptake in IL-2-maintained cells (Fig7G) shows that the concentration of Myc levels per cell dictates transferrin receptor expression and rates of transferrin uptake by immune-activated T cells. There is thus a clear biological consequence of fine-tuning Myc expression in single, activated T cells.

## Discussion

The expression of Myc in CD8^+^ T cells is regulated by both antigen receptor signals and the γc cytokine IL-2. The present study now shows that the TCR and IL-2 use distinct mechanisms to control Myc levels in T cells. The TCR controls Myc expression in a digital response that determines the frequency of the T cell population that switches on Myc mRNA and protein expression. In contrast, the cytokine IL-2 regulates Myc expression in an analogue response that fine-tunes Myc protein levels per cell. In the immediate response to TCR engagement, the strength of TCR signal controls how many cells express Myc, and at these time points there is no apparent role for IL-2/JAK signalling. Myc expression in T cells thus appears to be a two-stage process; Myc expression is digitally induced by the strength of TCR signalling, but the maintenance of high Myc expression then becomes dependent upon other external stimuli such as IL-2. Myc is essential for the metabolic reprogramming that controls effector T cell differentiation (Wang *et al*, 2011a). The fact that the strength of the TCR ligand determines the frequency of T cells that express Myc gives new insight about the mechanisms that allow TCR signalling strength to control the pool size of T cells that metabolically reprogramme and differentiate to effector cells.

One striking observation from the present study was the discordance between Myc protein and mRNA levels in immune-activated T cells. This latter observation is relevant because many previous studies have interrogated Myc mRNA expression in T lymphocyte subpopulations (Best *et al*, 2013). The data herein show that T cells can express high levels of Myc RNA but that high levels of Myc protein can only be sustained in T cells that have high rates of amino acid uptake. Myc expression in an antigen receptor-stimulated T cell will thus be transient unless the T cell can sustain high rates of biosynthesis of Myc protein. A key mechanism that controls Myc protein expression in activated T cells is mediated by the serine/threonine kinase GSK3. Hence, in activated T cells Myc is phosphorylated by GSK3 on Thr58, which then constantly targets the protein for proteasomal degradation (Gregory *et al*, 2003). It is this constant high rate of Myc degradation in activated T cells that confines high levels of Myc protein expression to T cells with high rates of amino acid uptake and protein synthesis. Myc protein expression thus rapidly declines in situations where T cell protein synthesis and amino acid uptake are limited. Recent studies have shown that there is very tight control of amino acid transport and hence protein synthetic capacity during the immune activation of T cells (Hayashi *et al*, 2013; Sinclair *et al*, 2013). The critical signals that are known to be able to induce and sustain high-level expression of amino acid transporters in T cells are antigen and pro-inflammatory cytokines such as IL-2 (Wang *et al*, 2011a; Sinclair *et al*, 2013). This means that Myc protein expression will be restricted to T cells responding to immune activation or to T cells exposed to pro-inflammatory cytokines that can maintain amino acid uptake.

One of the cytokines that can sustain high levels of Myc protein in T cells is IL-2. This molecule is a member of the γc family of cytokines that signal by activating the JAK tyrosine kinases. The present experiments interrogated the role of JAKs regulating Myc expression using the JAK inhibitor tofacitinib: a drug showing clinical promise for the treatment of autoimmune diseases (Hsu & Armstrong, 2014; Lee *et al*, 2014). Our data afford the insight that immune-activated T cells can express high levels of Myc mRNA and yet be dependent on sustained JAK activity to express Myc protein. Moreover, another novel insight from the present experiments is that part of the tofacitinib mechanism of action as an immunosuppressant may be to prevent T cell reprogramming of Myc-controlled metabolic responses. In the context of γc cytokines and JAKs, the strength of IL-2 signalling during an immune response determines effector versus memory cell fate of immune-activated CD8^+^ T cells (Kalia *et al*, 2010). Herein we show that IL-2 has the ability to sustain high rates of Myc expression compared to other γc cytokines such as IL-7 and IL-15, and this could contribute to the unique role for this cytokine as a regulator of peripheral T cell metabolism and differentiation. What is the mechanistic explanation for the differential effect of IL-2, IL-7 and IL-15? They all signal through the γc, and IL-2 and IL-15 share the same beta receptor subunit. Recent studies have shown that it is predominantly the abundance of the specific alpha chain for IL-2 and IL-15 that determines the strength of signal mediated by that cytokine (Ring *et al*, 2012). Furthermore, in T cells that highly express CD25, the alpha subunit for the high-affinity IL-2 receptor, signalling through IL-15 and IL-7 in particular is attenuated because of the sequestration of γc subunits in complete IL-2 receptors (Cotari *et al*, 2013). Therefore, even at saturating concentrations, IL-7 and IL-15 are less potent than IL-2 in our system.

The ability of T cells to rapidly fine-tune intracellular concentrations of Myc is striking but is it important? How sensitive are T cells to changes in Myc protein levels? We addressed this question by interrogating the importance of Myc protein levels for transferrin uptake. We show that Myc is essential for transferrin receptor expression in immune-activated T cells. Moreover, a salient result was that Myc protein levels determine the level of transferrin receptor expression and rates of transferrin uptake by immune-activated T cells. Myc is thus not an on/off switch for transferrin receptor expression but can fine-tune expression of this key receptor that ensures that T cells have sufficient iron. Myc has other roles in T cells, for example to control glucose and glutamine metabolism and to regulate expression of key transcription factors such as AP4 (Wang *et al*, 2011a; Chou *et al*, 2014). The present observation that there is coordination of Myc protein expression with amino acid uptake thus reveals a mechanism that would ensure that T cells coordinate increases in glucose and glutamine and iron metabolism to match the biosynthetic demands associated with the immune activation of T cells.

## Materials and Methods

### Mice and cell culture

All mice were kept in the Biological Resource Unit at the Wellcome Trust Biocentre, University of Dundee, in accordance with UK Home Office (Animals) Scientific Procedures Act 1986. Mice used were OT1 TCR transgenic mice, which express a TCR recognising the ovalbumin (OVA)-derived peptide SIINFEKL, or P14 TCR transgenic mice, which express a TCR recognising the gp33-41 peptide of LCMV (KAVYNFATM). GFP-Myc^KI^ was used as described (Huang *et al*, 2008). CD4Cre^+^ x Myc^fl/fl^ and control CD4Cre^−^ x Myc^fl/fl^ were used as previously described (Mycko *et al*, 2009). All mice are on a C57BL/6 genetic background and were used between 12 and 24 weeks of age.

For experiments involving T cell receptor (TCR) stimulation, CD3 antibody (2C11, 0.5 μg/ml) and CD28 antibody (clone 37.51, eBioscience, 3 μg/ml) were used for polyclonal T cell activation whilst OVA-derived peptides (10 ng/ml unless otherwise stated) were used for OT1 T cell activation for the specified times.

For experiments using cytotoxic T lymphocytes (CTL), cells were generated as described in Waugh *et al* (2009). Briefly, splenic CD8^+^ T cells from P14 mice were activated with 100 ng/ml gp33-41 peptide, washed and maintained with 20 ng/ml IL-2 (Novartis) or IL-15 (Peprotech) for a further 3–5 days, or in IL-7 (5 ng/ml, Peprotech) as indicated. Culture medium consisted of RPMI 1,640 medium containing L-glutamine (Invitrogen) with 10% heat-inactivated foetal calf serum (Gibco), penicillin–streptomycin (Gibco) and 50 μM β-mercaptoethanol (Sigma).

The proteasome inhibitor MG132 and GSK3 inhibitor CHIR99021 were synthesised by DSTT, University of Dundee. Tofacitinib (GSK) was used at 100 nM unless otherwise stated. Anti-mouse CD25 antibody (eBioscience, clone PC61.5) and IgG1 isotype control (eBioscience, clone eBRG1) were used at 2 μg/ml. For experiments involving T cell culture with the system L blocker 2-amino-2-norbornanecarboxylic acid (BCH, Sigma), cells were cultured in 50% RPMI culture medium and 50% Hanks’ buffered saline solution with or without 50 mΜ BCH.

### *Listeria* immunisation

An attenuated Act-A-deleted strain of *Listeria monocytogenes* that expresses OVA was used courtesy of Professor Hao Shen (Pearce & Shen, 2007). GFP-Myc^KI^ and WT control mice were immunised by intravenous injection of 5 × 10^6^ colony-forming units. Mice were culled after 24 h and spleens harvested for analysis.

### Retroviral transduction

The GFP control plasmid construct was made by PCR amplification of EGFP from a pEGFP construct (Clontech) and cloning into the pBMN-LZRS vector (Addgene) as a HindIII/NotI fragment, replacing the *lacZ* gene. Myc^T58A^ cDNA was generated by *in vitro* mutagenesis and the mutation confirmed by sequencing before cloning into the pBMN-I-GFP vector as an EcoRI fragment. Phoenix ecotropic packaging cells (Swift *et al*, 2001) were used to generate virus. The retroviral infection protocol of T cells was performed as previously described (Waugh *et al*, 2009).

### Flow cytometry and cell sorting

The following fluorochrome-conjugated antibodies (BD Pharmingen or eBioscience) were used for staining: CD4 (RM4-5), CD8 (53-6.7), TCRβ (H57-597), CD25 (PC61), CD44 (IM7), CD69 (H1.2F3), CD71 (C2F2), CD98 (RL388), IFNγ (XMG1.2), anti-GFP (rabbit polyclonal, Life technologies, for fixed samples in *Listeria* immunisation experiments) and Fc Block (2.4G2). DAPI was used as a live/dead discriminator.

Data were collected on FACSCalibur, Verse and LSR Fortessa machines (Becton Dickinson) and analysed using FlowJo software (Treestar). Fluorescence-activated cell sorting (FACS) was performed on a FACSVantage Cell Sorter (Becton Dickinson) to separate CD8^+^ GFP-Myc^pos^ and CD8^+^ GFP-Myc^neg^ live cell populations.

### Transferrin uptake assay

Cells were resuspended at 5 × 10^5^/ml in RPMI 5% BSA for 2 h prior to assay and then washed with RPMI 0.5% BSA before incubation with transferrin-alexa647 conjugate (Sigma, 5 μg/ml) at 37°C for 5 minutes. Uptake was stopped by washing twice with ice-cold acid wash (150 mM NaCl, 20 mM citric acid, pH 5.0). Transferrin uptake was measured by flow cytometry.

### Amino acid uptake

^3^H L-Phe or ^3^H L-Leu (Perkin Elmer) uptake into CTL was analysed using previously defined techniques (Sinclair *et al*, 2013), described in full in Supplementary Methods.

### Protein synthesis

CTL were re-suspended in methionine-free RPMI 1640 (Gibco Invitrogen) at 1 × 10^6^/ml. Triplicate samples were incubated in 96-well plates for 15 min before addition of ^3^H methionine (Perkin Elmer, 5 μCi/ml) and harvested after 15-min incubation at 37°C onto glass fibre plates (Perkin Elmer). Plates were soaked in HiSafe scintillant (Perkin Elmer) and incorporated ^3^H counted using a 1450 microβ+ counter (Wallac).

### Real-time PCR

CD8^+^ T cells were negatively selected from lymph nodes using AutoMacs (Miltenyi Biotechnology) prior to mRNA extraction. RNA was extracted from CTL or naïve cells using the RNeasy Minikit (Qiagen) and used to make cDNA using the cDNA synthesis kit (Quanta Biosciences). Quantitative real-time PCR (qPCR) was performed on an iQ5 (Bio-Rad) using SYBR Green Fastmix (Quanta Biosciences). The primers used are listed in Supplementary Methods.

### Western blotting

TCR-stimulated CD8^+^ T cells were purified by AutoMacs (Miltenyi Biotechnology) prior to protein extraction. Standard Western blotting protocols as described in Waugh *et al* (2009) were used. Blots were probed with antibodies recognising Myc, ERK1/2, pY694 STAT5, pan STAT5, GSK3α/β (Cell Signalling Technology), SMC1 (Cambridge Bioscience) and GFP (Roche Life Science). Densitometric analysis was performed using ImageJ software.

### Statistical analysis

Data sets were analysed using SigmaPlot v11.0 (Systat Software Inc., USA) or Prism 6. Comparisons between two groups were made using Student’s *t*-test or a non-parametric Wilcoxon rank-sum test where appropriate. Comparisons between multiple groups were made using one-way analysis of variance (ANOVA) test. Levels of significance are denoted as follows: * *P* < 0.05, ** *P* < 0.01, *** *P* < 0.001. Non-significant results are either not marked or indicated ns.

## Supporting Information

**Supplementary Materials and Methods**

**Review Process File**
