# Supplementary material for: Single cell tuning of Myc expression by antigen receptor signal strength and interleukin-2 in T lymphocytes
Source: EMBO J. 2015 Jul 1;34(15):2008–24. doi: 10.15252/embj.201490252 (PMC4551349; doi:10.15252/embj.201490252)
Supplement: Supplementary file 1 [file embj0034-2008-sd1.docx]

## Supplementary Information: Additional materials and methods

## Primers used for genotyping GFP-Myc^KI^ mice

GFP-Myc^KI^ mice were genotyped using the following primers:

5'-CGGACACACAACGTCTTGG-3' (forward)

5'‑TGAAGCTTACAGTCCCAAAGC-3' (reverse WT)

5'‑GTAGGTCAGGGTGGTCACGA-3' (reverse KI)

## Real-time PCR Primers

Primers for quantitative real-time PCR used were as follows:

Myc Forward: 5’-CCACCAGCAGCGACTCTG-3’

Reverse 5’-GAGATGAGCCCGACTCCG-3’

Slc7a5 Forward: 5′-CTGGATCGAGCTGCTCATC-3′

Reverse: 5′-GTTCACAGCTGTGAGGAGC-3′

CD8 Forward 5’-GATATAAATCTCCTGTCTGCCCATC-3’

Reverse 5’-ATTCATACCACTTGCTTCCTTGC-3’

IFNγ Forward 5'-TTACTGCCACGGCACAGTC-3'

Reverse 5'-AGATAATCTGGCTCTGCAGG-3'

CD25 Forward 5'-TTTCCTTCTGATCCCTGGGTTTC-3'

Reverse 5'-GATAGAGTTGCTGTTATGTCCTGTG-3'

CD71 Forward 5'-GACGCTTTGGGTGCTGGTGTTG-3'

Reverse 5'-GCCTGCAGTCCAGCTGGC-3'

CD98 Forward 5'-GAGGACAGGCTTTTGATTGC-3'

Reverse 5'-ATTCAGTACGCTCCCCAGTG-3'

PCR products were verified by DNA sequencing

## Western blotting

TCR stimulated CD8^+^ T cells were negatively selected from lymph nodes using AutoMacs (Miltenyi Biotechnology) prior to protein extraction. Cells were lysed at 3x10^7^/ml in 10mM Tris pH7.05, 50mM NaCl, 50mM NaF, 5μM ZnCl_2_, 1mM DTT, 10% glycerol and 0.5% Triton X100 with complete protease inhibitors (Roche). Lysates were separated by SDS-PAGE, transferred onto nitrocellulose membrane and detected by Western blotting using standard techniques. Antibodies used recognised Myc (cat number 9402), ERK1/2 (cat. number 9107), pY694 STAT5 (cat. number 9351), pan STAT5 (cat. number 9363), GSK3α/β (cat. number 5676) all from Cell Signalling Technology, SMC1 (cat. number A300-055A) from Cambridge Bioscience and GFP (cat. number 11814460001) from Roche Life Science.

## Amino acid uptake

1×10^6^ cells were resuspended in 0.4 ml Hank's balanced-salt solution (Gibco) containing ^3^H L-phenylalanine or ^3^H L-Leucine (0.5 μCi/ml) (Perkin Elmer) with a final extracellular L-leucine concentration of 5μM. Uptake was assayed for 4 min with samples layered over 0.5 ml of a mixture of silicone oil (Dow Corning 550 (BDH silicone products); specific density, 1.07 g/ml) and dibutyl phthalate (Fluka) at a ratio of 1:1. Cells were pelleted below the oil, then the aqueous supernatant solution, followed by the silicon oil–dibutyl phthalate mixture, was aspirated, and the cell pellet underneath was resuspended in 200 μl NaOH (100 mM). β-radioactivity was measured by liquid scintillation counting in a Beckman LS 6500 Multi-Purpose Scintillation Counter (Beckman Coulter). Each data point was performed in triplicate.

## Retroviral transduction

P14 splenic T cells were activated for 24 h with cognate peptide before centrifugation with freshly thawed retrovirus supernatant and polybrene (Sigma). Cells were cultured (37°C, 5% CO2) for a further 24 hours before centrifuging to remove polybrene and gp33-41. Cells were then resuspended in fresh medium containing IL-2 and cultured accordingly for a further 3-5 days.
