# Supplementary material for: Single cell tuning of Myc expression by antigen receptor signal strength and interleukin-2 in T lymphocytes
Source: EMBO J. 2015 Jul 1;34(15):2008–24. doi: 10.15252/embj.201490252 (PMC4551349; doi:10.15252/embj.201490252)
Supplement: Supplementary file 2 [file embj0034-2008-sd2.pdf]

THE EMBO JOURNAL

Manuscript EMBO-2014-90252

Single cell tuning of MYC expression by antigen receptor signal strength and Interleukin 2 in T lymphocytes

Gavin C Preston, Linda V Sinclair, Aneesa Kaskar, Jens L Hukelmann, Maria N Navarro, Isabel Ferrero, Hugh Robson MacDonald, Victoria H Cowling, Doreen A. Cantrell

Corresponding author: Gavin Preston, University of Dundee

Review timeline:

Submission date:	07 October 2014
Editorial Decision:	10 November 2014
Revision received:	03 April 2015
Editorial Decision:	06 May 2015
Revision received:	15 May 2015
Accepted:	18 May 2015

Editor: Karin Dumstrei

Transaction Report:

(Note: With the exception of the correction of typographical or spelling errors that could be a source of ambiguity, letters and reports are not edited. The original formatting of letters and referee reports may not be reflected in this compilation.)

1st Editorial Decision 10 November 2014

Thank you for submitting your analysis to The EMBO Journal. Three referees have now seen your study and their comments are provided below.

As you can see, the referees find the insights provided into the regulation of Myc in T cells interesting. However, they also find that much further analysis would be needed in order to solidify the conclusions and none of them are able to recommend publication here at this stage. Given the constructive comments provided, I can offer that should you be able to address the concerns raised in full that I would be willing to consider a revised version. However, I would also like to point out that I need strong support from the referees to move forward with the manuscript for publication here. If you find that you will not be able to address the concerns raised in full then it would be in your best interest to seek publication elsewhere at this stage.

I should remind you that it is EMBO Journal policy to allow a single major round of revision only and that it is therefore important to address the raised concerns at this stage.

Please bear in mind that this will form part of the Review Process File, and will therefore be available online to the community. For more details on our Transparent Editorial Process, please visit our website: http://www.embo.org/embo-press

Thank you for the opportunity to consider your work for publication. I look forward to your revision.

REFEREE REPORTS

Referee #1:

In this study, Preston and colleagues reported two distinct mechanisms regulating expression of transcription factor cMyc in CD8 T cells. The authors showed that while stimulation through the T cell receptor (TCR) increases Myc mRNA and more importantly "digitally" induces Myc protein expression. In contrast, signals via the IL-2Rγ exhibit an analog characteristic to fine-tune Myc levels primarily through enhancing nutrient uptake and translation. Based on these results, the authors propose the molecular mechanism by which activated CD8 T cells regulate Myc levels in response to extrinsic stimuli.

While it has been known that Myc expression is increased by TCR and IL-2R stimulation and that Myc protein expression levels are predominantly regulated by post-transcriptional mechanisms, distinct modes of regulation by TCR and IL-2R stimulation is described in detail for the first time in this work. Since the authors stated that IL-7 does not contribute to Myc protein expression, use of a term "gamma cytokine" is not appropriate. In addition, single concentrations of IL-15 and IL-7 compared to multiple concentration of IL-2 would not justify their conclusion that these three cytokines act differently. Otherwise, the authors used molecular biology and biochemistry to obtain consistent data throughout the paper. Additional experiments and use of appropriate control would be necessary to solidify their findings. Please see the following comments for the details.

Major points:
1. The authors concluded that the strength of TCR stimulation only affects the frequency of Myc-expressing cells but not the level per se (Fig. 1C, D). However, looking at the histogram overlays, it seems that N4 can induce higher levels of Myc than other peptides (top panel in Fig. 1C), and that increasing concentrations of peptide can enhance the level of Myc-GFP (bottom panel in Fig. 1C). The authors should provide statistical analysis on Myc-GFP peak values in the Myc-GFP+ population to validate their conclusion. Given that almost all the cells acquire Myc-GFP expression by 24 hours, what would be the biological significance of the "digital" induction of Myc? It may be helpful to separate GFP(+) and GFP(-) cells early after activation and examine expression of Slc7a5, which is necessary for Myc translation, and also culture them separately to see if there is any difference in proliferation and cytokine expression between the "early" versus "late" Myc expressers.
2. In Fig. 2E, the authors showed that treatment with JAK inhibitor led to a drop in Myc expression 24 and 48 hours after T cell activation, and suggested that autocrine IL-2 signaling contributes to regulation of Myc during TCR activation. However, JAK can be activated by pathways other than IL-2 signaling. To strictly test the effects of autocrine IL-2 on Myc expression, the authors should block IL-2 stimulation during T cell activation with anti-IL-2 neutralizing antibody. A more stringent way to address this question is to compare Myc expression in IL-2Ra+/+ and IL-2Ra-/- or IL-2+/+ and IL-2-/- CD8 T cells during CD3/CD28 activation.
3. In Fig. 3B, were the CD25+ cells activated by specific TCR stimulation or bystander activation, such as by IL-12 response by innate cells?
4. The authors argued that TCR stimulation induces Myc mRNA expression in a dose-dependent manner. However, in Fig. 4B, the qPCR analysis only revealed Myc transcript level in populations mixed with Myc+ and Myc- cells (Fig. 1). In addition, it is not clear how Myc mRNA expression relative to Hprt1 is calculated? Are data shown as relative to unstimulated cells? Values in each panel in Fig 4 are very different. This should be shown

© European Molecular Biology Organization

as raw values relative to appropriate controls. Also, cell sizes would be very different between different stimuli or treatment. Because Myc protein was measured by a per cell basis (GFP measurement in FCM), I feel that it would be appropriate to show Myc transcript levels based on a cell number normalized basis, such as by use of control RNA rather than with normalization to Hprt1, which might also change by different stimuli.
5. The authors used the data shown in Fig. 5E to argue that IL-2 stimulation increases Myc protein expression primarily through enhancing translation. To further demonstrate their point, the authors should compare the kinetics of Myc protein degradation in cells cultured with or without IL-2, in the presence a ribosome inhibitor, such as cycloheximide.
6. Fig. 1B, 3C and 3D should include appropriate controls using corresponding populations from WT mice without the Myc-GFP allele.
7. It is unclear how statistical analysis was done based on the Method. Please make sure that the analysis was done appropriately for multi-group analysis with ANOVA instead of t-test for individual permutation.

Minor points:
1. The authors argued that the effect of IL-2 on Myc expression correlates with Stat5 phosphorylation by JAK. The conclusion will be strengthened if the authors could observe a corresponding change in phospho-Stat5 in IL-2, IL-7 and IL-15 treated cells.
2. It will be interesting to see whether the requirement for IL-2 signaling to maintain Myc expression in vitro is also critical in vivo, for example, by comparing Myc expression in CD25+/+ and CD25-/- activated CD8 T cells.
3. Some methods are not described in details, which may affect reproducibility of data. For example, these studies used T cell/APC co-culture in many experiments, in which T cell to APC ratios would affect degrees of T cell activation. Also, although some experiments measured mRNA expression in T cells after co-culture (e.g, Fig 4B), it was not clearly described how T cells are separated from APC, so that the gene expression readouts should be consistent between independent experiments.
4. It is hard to distinguish different treatment conditions (Fig 1A, 7B etc.) in data presentation by histogram overlays.

Referee #2:

A picture is emerging whereby the regulation of metabolic programs is being seen as a critical component of T cell activation and differentiation. That is why this work regarding the regulation of Myc in T cells is both novel and important. Undoubtedly, the authors are on to something fundamental. Overall, while I agree with this line of inquiry and find it exciting I think that in many instances too broad a conclusion is being drawn from a nugget of supportive data. This is particularly true for the data regarding CD25 expression, CD71 expression and amino acid transport. Also, not enough detailed experiments are performed regarding the role of IL-2. Overall, what I am saying is that the authors should drill down in more detail to demonstrate more convincingly the mechanisms implied not just the associations. Please do not misinterpret my comments. I believe this work has hit upon something fundamental. I just think that more detailed mechanistic connections need to be defined. For example:

1. In Figure 1, how does the Myc expression relate in terms of CD69 expression? Have all the cells seen antigen yet only a subgroup express Myc? If so, what are the properties of the CD69+, Myc+ versus CD69+Myc- cels with regard to other markers of activation, effector versus memory markers, metabolism. Also, it would be interesting to see RNA levels for figures C and D

2. The authors make a strong case for the role of IL-2. But, what is the difference between IL-2 and IL-7 and Il-15 mechanistically. Is it simply degree of activation due to differential

© European Molecular Biology Organization

expression levels of the receptors? Also, no data is presented with other T cell subsets. Is the IL-2 dependence important for Th17 cells which are very glycolytic? If not why? What about Th1, Th2 and T reg cells. T regs would be of particular interest since they express high levels of CD25. But we are told they are not very glycolytic. What is the effect of tofactinib on specific metabolic function? Does it selectively inhibit glycolysis? Does it promote memory? What is the effect of this drug on the in vivo experiments focusing on Myc, metabolism and T cell differentiation.

3. The authors put a lot of faith in CD25 expression. However, as mentioned above how do T regs respond. Yes other activation markers don't correlate as well, but couldn't it be that CD25 expression is a marker for a specific type of activation program which may include Myc regulation as well as many others that are relevant to the model

4. The GSK inhibitor is nice as well as the mutant Myc resistant to degradation. Can these reagents by explored further to demonstrate their effect on overall metabolism and differentiation. Exploring this further would be more powerful than the very narrow (though convincing) evaluation of CD71

5. It is not that I think amino acid transport is irrelevant but I think that more convincing experiments need to be done to prove the Production over degradation model. Some classic protein stabilization experiments and the use of inhibitors of protein synthesis might help develop this model better.

6. From reading the literature it seems that a lot of the observations might be explained by differential mTOR activation (decreased leucine import, CD71 and even the regulation of Myc itself). How does this fit in.

Referee #3:

In this submission the authors aim to dissect the contribution of antigen receptor-mediated signals and cytokine driven activation in regulating Myc expression and function in T cells. The question is relevant and the methods applied suitable to address the issue. The manuscript is generally well written. However, a number of concerns somewhat dampen enthusiasm for this manuscript.

The key claims of the authors are (i) that antigen receptor mediated signaling provides an on-off signal for Myc transcription, whereas cytokine receptor signaling fine tunes Myc via post-transcriptional control, and (ii) that Myc expression matches the biosynthetic needs of T cells. Based on the data provided this seems too big a statement. In the experiments that aim to dissect TCR vs. cytokine signaling in regard to Myc expression, sometimes anti-CD3/CD28 activation is used (which is combining TCR signaling and co-stimulation) sometimes peptides are used (incl. peptide variants) -which is a very different setting- and yet these experiments are together used to make the argument that TCR signaling (!) is providing a distinct on-off mechanism with regard to Myc transcription. This is confusing and needs to be experimentally addressed. Even if the above issue is solved (TCR +/- CD28 co-stim. vs. peptide activation vs. cytokine-mediated co-stimulation) it remains unclear how the concept of an on-off switch provided by TCR signaling is compatible with the approx. linear correlation (= clearly not an on-off phenomenon) of GFP-Myc expression and CD25 expression, and the very tight link between Ag dose/affinity and IL-2 production firmly established in the literature (see also below).

Regarding the biosynthetic needs no data are shown that link nutrient uptake and actual biosynthesis (i.e. proliferation (Myc expression in proliferating vs. non-proliferating cells?); production of cytokines; production of cytolytic proteins, etc.) -making it

impossible to relate nutrient uptake-data, Myc expression and biosynthesis.

Throughout the manuscript it is quit hard to know what really has been done, since very little precise information is provided in the text, the figure legends and the figures themselves (incubation times, types of experiments (WB vs. RNA asessments, etc.), quantification of WB data, actual data-points (rather than bar graphs summarizing as little as 3 data points), etc.).

Specific major concerns in relation to each of the figures are listed below:

Figure 1: Myc-expression in CD8 T cells follows a threshold-phenomenon regulated by peptide affinity which can be compensated for, in part, by peptide dose.
(i) % of Myc expressing cells correlates with peptide affinity and peptide dose. Compared to non-specific activation (CD3/CD28) not 100% of cells become Myc-positive (or is this a time-point issue? Please show data for the 24h time-point). Thus, what happens to the Myc-non-expressors? Do they dye off, etc.
(ii) According to published literature, peptide affinity and peptide dose are correlated with IL-2 production and CD25 expression (Zehn 2009, Kalia 2010, Tkach 2014). Given the data in Figure 2, how do the authors explain the on/off phenomenon shown here? How does Myc-expression look like at 24h post-activation?
(iii) By 72h hours, Myc-expression starts to decrease. Is this due to cell proliferation or decrease in IL-2 levels?
(iv) In Fig 1D it is unclear whether CD3 only or CD3 plus CD28 activation was used. Also, the concept of TCR mediated signaling being an on off switch is not supported by the fact that co-stimulation seems to increase the percentage of pos. cells (contribution of TCR vs. CD28 co-stimulation).

Figure 2: The level of activation-induced Myc-expression correlates with the strength of IL-2 (and in part IL-15) mediated JAK-STAT signaling.
(i) 4h post-activation, almost all cells express Myc (which is different from Figure 1B). Abrogating IL-2 signaling does not have an impact on Myc-expression, whereas by 24h post-activation it does. It would thus be interesting to elucidate the effect of JAK-inhibition on different peptide affinities and doses. Does the threshold-phenomenon apply here too?
(ii) How do the mRNA data in Figure 2A relate to Myc expression? It would be nice to see eGFP-Myc expression (FACS and Western Blot) upon IL-2 treatment +/- peptide stimulation.
(iii) Only 5ng/ml for IL-7 vs. 20ng/ml for IL-2 and IL15: why? A dose response for all three cytokines is required (throughout) to relate these data to the biology of each of these cytokine's effect.
(iv) In OT-I cells there is a two-population distribution between Myc pos. and neg. populations at 4h post activation, whereas in P14 CTL at the same time point there is only on 100% MYC pos. population. Please explain/address.

Figure 3: Myc-expression correlates with CD25 expression, yet this seems to argue against the claim that CD3/TCR signaling provides an on/off signal.
(i) Reg. panel D: if gating on CD25 very high vs. high vs. pos: Myc expression?

Figure 4: Myc mRNA levels correlate with peptide affinity but are independent of IL-2-JAK-signaling.
(i) Are the cells in C-E activated or resting? Please provide more details (activated yes no, if so: with what and for how long, etc)? (See also above (general comments) reg. clarity of figures and sufficiency of information provided).

Figure 5: Myc mRNA levels are independently expressed of IL-2, whereas Myc transcription is IL-2 dependent. Myc is rapidly phosphorylated and degraded even in the

presence of IL-2.
(i) The sentence "Figure 5A shows that T cells transduced with the Myc-IRES-GFP vector overexpress Myc mRNA independently of the presence or absence of IL-2" is misleading, since in Figure 5A Western Blot data are shown.

Figure 6: IL-2 mediates upregulation of amino acid transporters in a JAK-dependent manner to maintain Myc-levels
(i) What is the toxicity of Tofacitinib on T cells?
(ii) How specific is this for Myc? Is protein synthesis generally reduced? Please provide additional read outs and information.
(iii) Dose response for cytokine-responses (as stated above)

Figure 7: Myc regulates expression of CD71 and transferrin uptake.
(i) What is the effect of Tofacitinib on CD69, CD62L, CD44 expression?
(ii) Matching metabolic need: please substantiate (see general points above).

1st Revision - authors' response 03 April 2015

Referee #1:

We would like to thank this reviewer for recognising the novelty of our work on Myc expression both in terms of its impact for the immunology community and the new information it will provide to the global community of biologists who study Myc expression. This concept of digital control of Myc expression is a novel insight as is the recognition that a critical cytokine IL-2 can fine-tune Myc expression through enhancing amino acid uptake.

The IL-2 receptor is a member of the common gamma chain family of cytokine receptors. The reviewer is correct that we have not looked at all members of this family so we have amended the paper to make this point. In this regard the reviewer queried why we used single concentrations of IL-15 and IL-7 compared to multiple concentration of IL-2. We apologize for not explaining this choice. When we compare the actions of the three cytokines we use single doses of cytokine that fully saturate the respective cytokine receptors. Increasing levels of any of these cytokines beyond this level has no further biological effect.

In the context of IL-2, we wanted to understand how analogue the IL-2 response was and hence the decision to titrate this cytokine to see the impact of IL-2 receptor occupancy on levels of Myc expression.

Specific points

1) As suggested by the reviewer we have modified Fig 1 to more quantitatively illustrate the point that the strength of TCR stimulation with different physiological TCR agonists influences the frequency of T cells that express GFP-Myc but not the amount of GFP-Myc expression per cell. We now show combined data from multiple experiments rather a single representative data set.

2) The reviewer questioned the biological significance of the "digital" induction of Myc? As suggested by the reviewer we used FACS to separate GFP(+) and GFP(-) cells early after TCR activation with peptide to examine expression of Slc7a5 . We found that the GFP Myc positive cells had higher levels of expression of Myc mRNA and higher levels of expression of mRNA slc7a5 and its dimer partner CD98. We also found higher levels of transferrin receptor expression and Interferon gamma production in the GFP-Myc positive

© European Molecular Biology Organization

cells. We further verified that cells that express cMyc make higher levels of Interferon gamma mRNA. These data are now included in the manuscript. We also show it is the TCR activated T cells that express Myc undergo blastogenesis.

3) In the context of the biological significance of the digital induction of c-myc it is important to stress that with a very polyclonal TCR agonists such as high concentrations of CD3 antibodies or very high concentrations of peptide /MHC complexes then the digital/bimodal Myc expression is only seen at the early time points. With lower levels of CD3 antibodies or importantly, with lower affinity TCR physiological TCR agonists or lower doses of peptide/MHC complexes the digital nature of the response is sustained. We now include new data in Fig 1 that address this point. In particular much more data of how T cells respond to sustained signaling with different TCR agonists. The paper also includes new data in Fig 1 and Fig 6 that explore the functional consequences of bimodal Myc expression.

4) In the context of cells responding to high doses of high affinity TCR agonists we have thought very long and hard about how to test the importance of the early versus later Myc expression and cannot think of a good experiment. However, in experiments to address this question we used FACs to sort the Myc expressing and non-expressing T cells at the early time points and we now know that the cells that express high levels of Myc at an early time point also have high levels of Slc7a5 and CD98 and high levels of Interferon gamma. The new experiments thus show that there is a bi model induction of Slc7a5 expression. This transporter is important for not only for Myc expression but also mTOR activity and for expression of glucose transporters. We would thus never know if any differences in the Myc positive or Myc negative cells were due to the Myc expression or differences in Slc7a5 expression. We do know that if we delete Myc then T cells do not response to TCR triggering so the importance of Myc expression is unequivocal.

5) As suggested by the reviewer we did experiments with IL-2 neutralizing antibodies to examine the role of autocrine IL-2 production on the expression of Myc in response to TCR triggering of CD8 T cells. We do not have the IL-2Ra-/- or IL-2-/- mice in Dundee but the experiments with IL-2 neutralizing antibodies provided valuable new information. We thank the reviewer for this positive suggestion. The data show that an IL-2 neutralising antibody reduced the levels of Myc expression in TCR activated T cells. These data are now shown in Fig 2E. The salient point is that Myc expression is not sustained when IL-2 signaling is blocked. The IL-2 neutralising antibody did not change the frequency of activated T cells that expressed Myc but reduced the amount of Myc per cell. This strengthens our conclusion that IL-2 controls the expression of Myc in an analogue fashion.

6) The reviewer questioned whether the CD25+ cells detected in spleens of Listeria infected mice were only responding to specific TCR stimulation or influenced by bystander activation, such as by an IL-12 response by innate cells? This is a question that is almost impossible to answer as in vivo there will always be an influence of bystander activation. In the context of IL-12 then of course naive T cells do not express IL-12 receptors and so any IL-12 effect still needs TCR engagement to induce expression of IL-12 receptors. It is our experience that if one just injects LPS to mimic bystander stimulation only then we do not see CD25 expression by CD8 T cells although the cells will express CD69. I have worked on the signals that control CD25 expression in naïve CD8 T cells for many years and it seems that the key trigger initial trigger is the antigen receptor and that bystander effects come in on the TCR activated cells. There will no doubt be a contribution of IL-12. However, the experiments in Fig 3 are not addressing what signals control expression of CD25, they are exploring the idea that high levels of CD25 expression mark T cells that express high levels of Myc.

7) The reviewer made the point that Fig 4B showed qPCR analysis of Myc transcript level in TCR stimulated populations mixed with Myc+ and Myc- cells. We apologise because

we did not effectively explain this experiment but what we have done is re-organise the paper and explain the rationale for the experiments more thoroughly. We also have new data in Fig 1 that show that only cells that express Myc protein express Myc mRNA. We feel that this reorganization and rewriting of the paper improves the manuscript.

8) As suggested by the reviewer we have re-analysed mRNA data throughout to show Myc transcript levels normalised on a per cell basis to a specified control within each Figure. For example, in Figure 4B-C the control is naïve CD8 T cells whereas in Figures 4D-F it is IL-2 cultured CTL.

9) The reviewer suggested that an experiment to further support our idea that IL-2 stimulation increases Myc protein expression primarily through enhancing translation would be to compare the kinetics of Myc protein degradation in cells cultured with or without IL-2, in the presence a ribosome inhibitor, such as cycloheximide.

Our response is that this type of experiment with protein synthesis inhibitors can be complicated to interpret because these compounds activate the p38 MAP kinase. Moreover, as a postdoctoral fellow in 1985 I showed that the high affinity IL-2 receptor has a very short half life in the presence of protein synthesis inhibitors (Smith & Cantrell, 1985). Hence IL-2 plus cycloheximide is basically like withdrawing IL-2. We do however feel that there is constant proteolysis of Myc in IL-2 maintained cells equivalent to no IL-2. We agree that we cannot exclude entirely an increase in Myc proteolysis on IL-2 withdrawal. Nonetheless, there is clearly active proteolysis with IL-2 present since inhibition of the proteasome rapidly increases Myc expression even in the presence of IL-2 (Figure 5E). Hence the only way to sustain Myc levels would be to synthesize new Myc protein. Note we have not meant to imply that IL-2 controls Myc translation rather that IL-2 controls high levels of amino acid uptake and this is needed to keep Myc levels high in T cells. We now include new data in Figure 5 that show the effects of blockade of the System l transporter causes loss of Myc protein. Hence we feel that we show that T cells need continual high rates of amino acid uptake to sustain high levels of Myc protein.

10) The reviewer asked that Fig. 1B, 3C and 3D should include appropriate controls using corresponding populations from WT mice without the Myc-GFP allele. We have modified figures to include control data as appropriate. Note the order of the figures is now changed to present the data in a more logical fashion.

11) We apologise that there was a lack of clarity about our statistical analysis. We would like to confirm that all multi-group analysis was performed with ANOVA and not t-test.

We have addressed the minor points made by the reviewer and in particular have changed the figures to get better clarity between the histograms as suggested. We have also edited the materials and methods and Figure legends to clarify the experimental details used for each experiment. The paper has been edited and rewritten to make our experimental strategy clearer to the reader.

Referee #2:

We thank the reviewer for many interesting questions. We have revised the paper to include more experiments that address many of the issues raised. One of the key points we wanted to make but perhaps did not emphasize sufficiently is that the TCR is a digital switch for Myc mRNA and protein expression that allows the strength of the antigen stimulus to determine the frequency of T cells that switch on Myc expression. IL-2 signaling strength also directs Myc expression but in an analogue process that fine tunes Myc quantity in individual cells. It shows that there can be two quite different ways to

control Myc at the population level and at the level of an individual cell. I think the combining of digital and analogue processes allows tight control of Myc expression at the population and single cell level during immune responses. We have used the TCR and IL-2 as these are key signals. We did not however mean to imply that these would be the only stimuli that can control Myc levels. We have included new experiments and new analyses that should clarify the issues raised by the reviewer.

1) The reviewer questions how Myc expression relates to CD69 expression? Have all the cells seen antigen yet only a subgroup express Myc? We now include data that address this point. The manuscript has been reorganized and the relevant data are shown in Fig 1 and Fig 6. The relevant data are the experiments that compare the antigen dose for CD69 and Myc expression. We now include data showing that the antigen dose response for CD69 and Myc induction are different - CD69 induction occurs at a lower threshold of TCR triggering. It is possible to have an antigen receptor trigger where only a subset of CD8 cells that have upregulated the activation markers CD69 will express Myc. The CD69+ve cells expressing high levels of Myc are larger than CD69 high, Myc low cells indicating that they are the ones that have undergone blastogenesis. We also show that in the immediate response to TCR engagement it is only Myc high cells that express Myc mRNA . These data show that the TCR induces a digital response of Myc RNA and protein expression.

2) The reviewer questions the mechanistic difference between IL-2, IL-7 and IL-15 and asks if it is simply degree of activation due to differential expression levels of the receptors? We have previously addressed this issue in the context of IL-2 and IL-15 (Cornish et al Blood 108: 600-608). Moreover it is well established that low dose IL-2 has the same biological effect as saturating IL-15. We also know that TCR activated effector T cells express <1000 IL-7 receptors and approximately 10,000-15,000 high affinity IL-2 receptors. Moreover, Furthermore, in T cells that highly express CD25, the alpha subunit for the high affinity IL-2 receptor, signalling through IL-15 and IL-7 in particular is attenuated because of the sequestration of γc subunits in complete IL-2 receptors (Cotari *et al*, 2013). We have modified the manuscript to explain these differences.

3) The reviewer questions what happens to Myc expression in other T cell subsets? This is indeed an interesting question but a vast question and beyond the scope of the present study. We agree with the reviewer that regulatory T cells are an interesting population of cells. They do express high levels of CD25 but it is clear that IL-2 does not signal in regulatory T cells in the same way that it signals in CD8 effector cells or in T_H1 cells. The reason why Tregs signal differently has been ascribed to their expression of high levels of the negative regulator PTEN but is likely to be more complex.

4) The reviewer asks about the effect of Tofacitinib on specific metabolic function in T cells? The effects of this drug are complex and a study in itself. JAK inhibition has multiple effects on T cell metabolism and there is no room in the current paper to include these data. As predicted from its effects on Myc we see loss of glucose transport and glycolysis and also glutamine uptake but it also controls mTOR and STAT activity independently of any effect on Myc and we hence see loss of lots of metabolic pathways. We have not done in vivo experiments with this drug although it is used clinically as an immunosuppressant.

5) The reviewer is correct that our data that cells with high levels of CD25 have high levels of Myc is correlative. However, the manuscript now includes experiment with IL-2 neutralising antibodies that support that IL-2 controls Myc levels per cell rather than the frequency of cells that express Myc.

6) We have included further experiments with the GSK3 inhibitor that illustrate that inhibiting this kinase causes cells to increase levels of transferrin receptor. We have not expanded the study to look at the role of GSK3 in regulating further aspects of metabolism. The paper as it stands represents about 4 years of work by a team and represents a large body of work. We appreciate that our study raises other questions but we feel we have a substantial body of work here.

7) As suggested by the reviewer we did consider experiments with protein synthesis inhibitors to probe Myc turnover. There are two problems. First protein synthesis inhibitors activate the p38 MAP kinases which control protein translation. This complicates interpreting their effects. Second, as a postdoctoral fellow in 1985 I showed that the high affinity IL-2 receptor has a very short half life (approximately 15 mins in the presence of protein synthesis inhibitors (Smith & Cantrell, 1985). Hence IL-2 plus cycloheximide is basically like withdrawing IL-2. We do however feel that there is constant proteolysis of Myc in IL-2 maintained cells equivalent to those with no IL-2. We agree that we cannot exclude entirely an increase in Myc proteolysis on IL-2 withdrawal. Nonetheless, there is clearly active proteolysis with IL-2 present since inhibition of the proteasome rapidly increases Myc expression even in the presence of IL-2. Hence the only way to sustain Myc levels would be to synthesize new Myc protein. Note we have not meant to imply that IL-2 controls Myc translation rather that IL-2 controls high levels of amino acid uptake and this is needed to keep Myc levels high in T cells.

8) We have previously explored the link between mTOR and Myc expression and found no role for mTOR in controlling the expression of Myc (Finlay et al J exp med 2012, Sinclair et al Nature Immunology 2013). We have now included some discussion of this and the appropriate references to clarify this point.

Referee #3:

We thank the reviewer for the useful comments about the paper and we have revised the manuscript and included new experiments to address the points raised. We have thoroughly edited and reorganized the paper to make it clear what experiments were done and how T cells were activated in the different experiments. The data in this paper represent a huge body of work and there is the dilemma of how to present large amounts of data e.g. whether to show representative experiments or to combine data from multiple biological replicate experiments. We have tried to do the latter as we feel it is more balanced. We have re-written all figure legends for clarity. We apologise that the paper was not easy to follow.

1) We apologise for the confusion in the way we presented the data about the antigen receptor and digital control of Myc expression. The most appropriate way to probe this is with peptide/MHC complexes as TCR ligands as these are the physiological ligands for the TCR. We now show these peptide stimulation data first and include more experiments with different ligand concentrations. We also include experiments with CD3 and CD28 antibodies and what we have now made clear is that when we titrate the CD3 antibody we keep CD28 levels (costimulation) constant. We also used FACS to separate GFP(+) and GFP(-) cells early after peptide triggering of TCR complexes and find that the GFP Myc positive cells expressed Myc mRNA compared to the negative cells consistent with digital induction of Myc mRNA and protein in the immediate response to TCR ligation. These data show that antigen receptor mediated signaling provides an on-off signal for Myc transcription.

2) We agree with the reviewer that the links between antigen receptor signaling and IL-2 signaling can be complicated to unravel. There is a very tight link between Ag dose/affinity

© European Molecular Biology Organization

and IL-2 production. However, it is well established that the level of TCR triggering required to induce IL-2 receptor expression i.e. IL-2 responsiveness is lower than that required for IL-2 production so the frequency of cells that express the IL-2 receptor will exceed the number of cells that make IL-2. Thus secreted IL-2 can act on T cells that do not make their own IL-2. The reported polarised production of cytokine may mean that at certain stages of the activation response IL-2 works very locally. It is also pertinent that other signals can sustain IL-2 receptor expression in effector T cells. eg TCR triggering is needed to switch in IL-2 and IL-12 receptor expression but IL-12 can then drive further IL-2 receptor expression. Hence IL-2 responsiveness can be sustained for several days following the loss of the TCR ligand. IL-2 made by other cells can thus work on these IL-2 responsive T cells.

Our data do show clearly that TCR signaling cannot sustain Myc expression and levels will drop off. At this point the ability of IL-2 to sustain Myc levels become relevant. This point is illustrated by a number of facts. First, the approx. linear correlation of GFP-Myc expression and CD25 expression is only seen in the sustained response to TCR triggering i.e. after 24 hours. Second, the JAK inhibitor only decreases Myc levels in the sustained and not the immediate response to TCR triggering. Third, we now include data with IL-2 neutralizing antibodies and again these only decrease Myc levels per cell in the sustained response and not in the immediate response. Note at no point do we see that IL-2/JAK signaling controls a digital response and IL-2/JAK signaling can clearly control Myc protein without controlling Myc mRNA.

We do feel out data shows Myc expression in T cells can be regulated by two distinct mechanisms. Myc mRNA and protein expression are digitally induced by the strength of TCR signalling but maintenance of high Myc expression then becomes dependent upon ongoing γc cytokine signals, notably IL-2. Myc is essential for the metabolic reprogramming that controls effector T cell differentiation. The digital nature of Myc induction by the TCR is thus a mechanism that would control the pool size of T cells that can metabolically reprogram and differentiate to effector cells. We hope the extra experiments and the re-organisation of the paper now clarify the points raised by the reviewer.

3) The reviewer was concerned that we had shown no data to link Myc to actual biosynthesis. We now include data in Fig 6 showing that activated T cells that express Myc are bigger than activated T cells with no Myc expression. We were also relying and referencing the excellent study from Doug Green's group showing that Myc controls multiple metabolic ie biosynthetic pathways in T cells.

We also now show that activated T cells expressing Myc make much more Interferon gamma than those with low levels of Myc in the same cultures. It is clear that only T cells that express Myc protein have high levels of amino acid uptake and our previous data has shown that in the absence of amino acid uptake T cells cannot blast or proliferate (Sinclair et al 2013).

4) We have included new data showing the effect of different peptide affinities and doses at different time points as suggested by the reviewer. We have not followed what happens to the Myc null cells in culture.

5) The reviewer is correct that peptide affinity and peptide dose are correlated with IL-2 production and CD25 expression (Zehn 2009, Kalia 2010, Tkach 2014). We have discussed the two stage process for Myc regulation where the cytokine response becomes relevant in later time points. The underlying mechanism is that initially TCR induction of expression of amino acid transporters is digital and IL-2 independent. It is during the sustained

response (later time points) to TCR triggering with either peptides or CD3 antibodies when an autocrine IL-2 response becomes relevant.

6) The reviewer questioned whether the decreases in Myc-expression in 72 hour CD3/CD28 activated T cells are due to decreases in IL-2 levels. We would think it was. First addition of IL-2 will restore Myc levels. Second IL-2 neutralizing antibodies decrease Myc levels even further in these cells. The inclusion of the experiments with the blocking IL-2 antibodies allows us to discuss this point.

7) We have modified the figure legends and revised the manuscript to more clearly describe the experiments with CD3 and CD28 antibodies. Note in these experiments we titrated CD3 but kept CD28 antibodies constant.

8) We have revised the paper to illustrate more clearly the effect of peptide affinity and IL-2 on Myc mRNA versus protein.

9) The reviewer queried why we used 20ng/ml of IL-15 and 5ng of IL-7 compared to multiple concentration of IL-2. We apologize for not explaining this choice. When we compare the actions of the three cytokines we use single doses of cytokine that fully saturate the respective cytokine receptors. Increasing levels of any of these cytokines beyond this level has no further biological effect.

In the context of IL-2 we wanted to understand how analogue the IL-2 response was and hence the decision to titrate this cytokine to see the impact of IL-2 receptor occupancy on levels of Myc expression.

10) The reviewer asked us to explain why in OT-I cells there is a two-population distribution between GFP Myc pos. and neg. populations at 4h post activation, whereas in P14 CTL at the same time point there is only on 100% MYC pos. population.

Our response is that we have never made P14/GFP-Myc mice only OT1/GFP-Myc mice. The confusion came from the brevity of our figure legends and we have now corrected these. We frequently use the P14 TCR transgenic model but only to produce fully differentiated effector cytotoxic T cells because this is a very well characterized model to generate CTL and to study IL-2 signaling in CTL. Note that IL-2 controls Myc expression in CTL generated from OT1 mice or in polyclonal CTL generated by activating T cells with CD3/CD28 antibodies followed by expansion in IL-2.

11) The reviewer questions the toxicity of Tofacitinib on T cells? We can assure the reviewer that this drug is not toxic, it does not kill CTL rather it is cytostatic. Our data with Myc have prompted us to explore in detail the impact of this drug on the T cell proteome. Using mass spectrometry we identified 7000 proteins in IL-2 maintained CTL. Treatment with Tofacitinib even for 24 hours had quite selective actions and decreased expression of about 191 proteins but increased expression of 128 proteins. So there is no global effect on protein synthesis rather a reshaping of the T cell protein landscape. This is a paper in its own right and to include this data would be beyond the scope of the present paper.

2nd Editorial Decision 06 May 2015

Thank you for submitting your revised manuscript to The EMBO Journal. Your manuscript has now been re-reviewed by the referees and their comments are provided below.

As you can see, the referees appreciate that the added data and are supportive of publication here. Referees #2 and 3 have some remaining comments that would be good to

sort out before acceptance of the paper. I think that most of the remaining issues can be resolved without further experiments, but with better clarification. Let me know if we need to discuss some of the issues further.

You can upload the revised version using the link below.

REFEREE REPORTS

Referee #1:

The authors have thoroughly addressed the concerns raised by the reviewers either by clarification or adding experimental data. Now I feel that the authors' conclusion in the manuscript is well supported and this work provided insights into differential cMyc regulation by TCR and IL-2R signals.

Referee #2:

In this report the authors dissect the expression of Myc upon antigen recognition. This work provides detailed insight into expression of Myc (in a digital fashion) as regulated by TCR signal strength and then the fine tuning of Myc by IL-2 signaling. For the most part the authors have experimentally addressed my concerns. Still though in light of the role TCR signal strength in directing T cell differentiation (Th2 cells, T regs, anergy) it would have been nice to have seen the consequences of Myc expression in relation to these different outcomes. Likewise, the robustness of the true significance of the correlation with CD71 expression is not clear since CD71 is regulated by multiple pathways (including mTOR). Nonetheless, overall the data provide detailed insight into the events that regulate Myc in T cells and thus provide a strong foundation for the further study of this important regulator of T cell metabolism.
One minor note. It might be more informative to view the data in Figure 3b and C relative to no IL-2. This way you can see not only the lack of difference between the cytokines or in the presence of the drug but also the magnitude of the IL-2-induced increase.

Referee #3:

Preston et al., EMBO re-submission

General comment: the authors have carefully addressed most of the points raised and provide new data that significantly improve the manuscript. However, a few points remain to be answered:

1. The authors include now data using IL-2 neutralizing antibodies, and claim that these only decrease Myc-expression in the sustained and not the immediate response. However, only data at 48 hours post-activation is included and it is not clear, whether IL-2 neutralizing antibodies would also decrease the population of Myc-expressing cells at early time-points (i.e. the digital response).

2. It is still not clear what happens to the Myc-negative population. Since many of these cells do seem to be activated (CD69+), and to some extend also blasted (Fig. 6), it would be interesting and experimentally easy to follow this population.

3. Myc expression seems to be relevant for immediate (2h) but also sustained (24h) IFNg production. However, the authors do not show that IFNg is actually secreted, which is of

functional relevance. Also it would be interesting to know, whether the amount of Myc per cell is correlated to the amount of IFNg the cell produces (fine-tuning).

4. Myc-expression was decreased when blocking AA-uptake or putting cells in AA-deprived medium. Is this in any way specific to Myc, or is protein synthesis simply reduced overall?

5. The relevance of the correlation between Myc-expression and transferrin uptake is not clear to me. In order to add some specificity to the effect(s) of Myc it would be of particular interest to see something that does not decrease without Myc, and -as stated above- what happens to the Myc-negative population.

2nd Revision - authors' response 15 May 2015

We have worked hard to revise the paper and include new data that address the issues raised by the reviewers. We feel that we have addressed the majority of the issues. There was one we felt was unreasonable e.g. our paper focused mostly on effector CD8 T cells with some data about CD4 effector cells. The reviewer wanted to know what happened to regulatory T cells. We agree this is an interesting question but beyond the scope of the present paper. I apologise that this has taken us such a long time but I think the revised paper is much improved and we hope it is now acceptable for publication in EMBO. We feel it gives important insights about how two key immune-modulatory signals, antigen and the cytokine IL-2, can co-ordinate a tight control of Myc expression at the single cell level. There is an increasing interest in single cell transcriptome analysis and our data about quantitative difference in Myc expression at the single cell level and the consequences of this are timely. We show the mechanistic basis for this ability to fine tune myc levels in single cells is controlled by rates of amino acid uptake by cells which show how cells coordinate their responses to match their metabolic states. Note the reworking of the paper has made us modify the title to more accurately reflect what the work shows and we hope this is appropriate.

© European Molecular Biology Organization
