# Supplementary figures and images for: Single cell tuning of Myc expression by antigen receptor signal strength and interleukin-2 in T lymphocytes
Source: EMBO J. 2015 Jul 1;34(15):2008–24. doi: 10.15252/embj.201490252 (PMC4551349; doi:10.15252/embj.201490252)

Figure 1 A

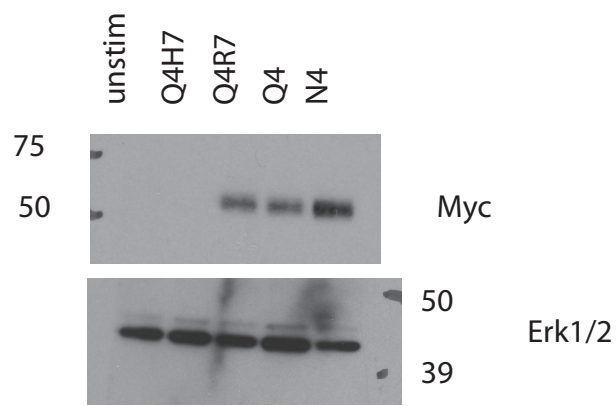

Supplement: Supplementary file 3 [file embj0034-2008-sd3.pdf]

2A

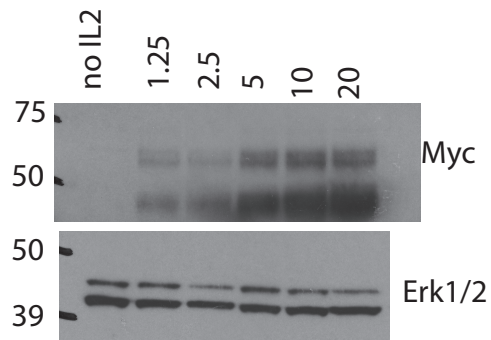

2B

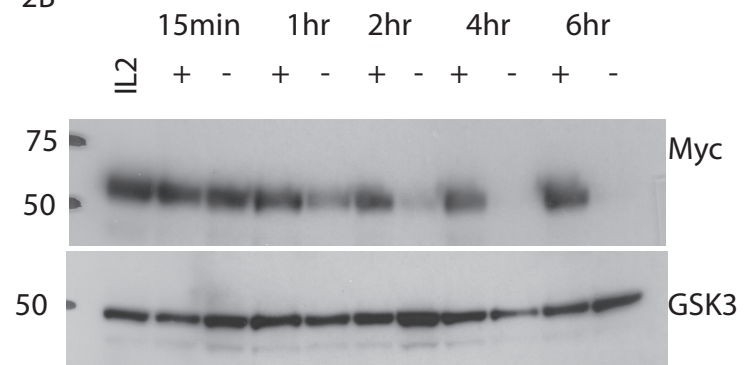

2C

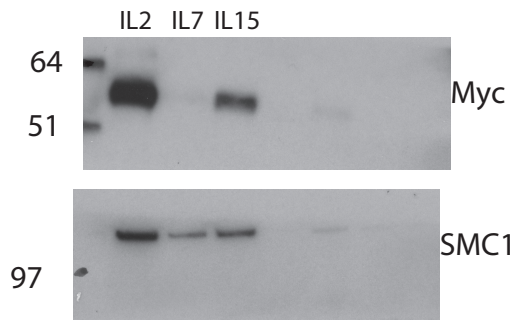

2D

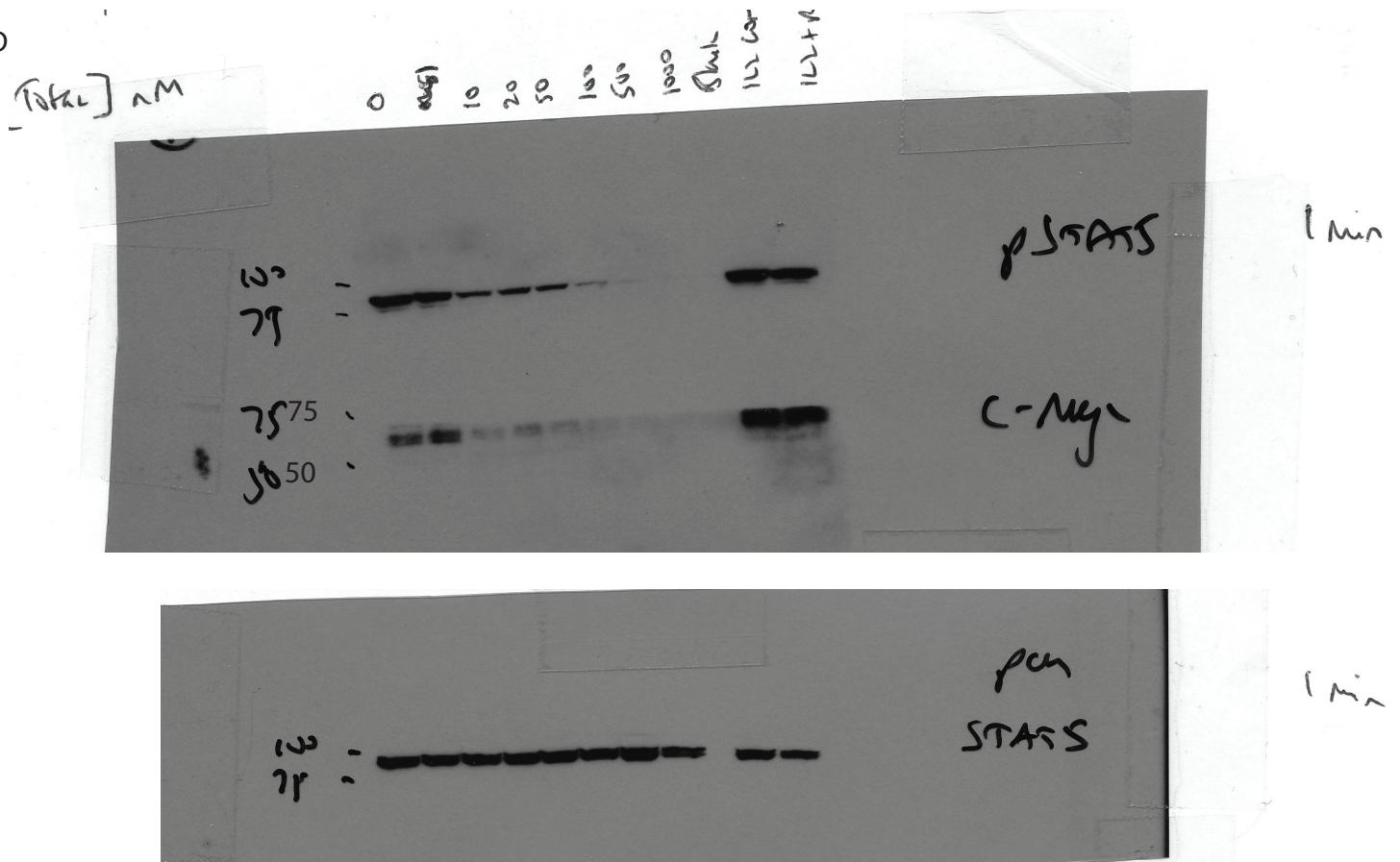

Supplement: Supplementary file 4 [file embj0034-2008-sd4.pdf]

Figure 3D

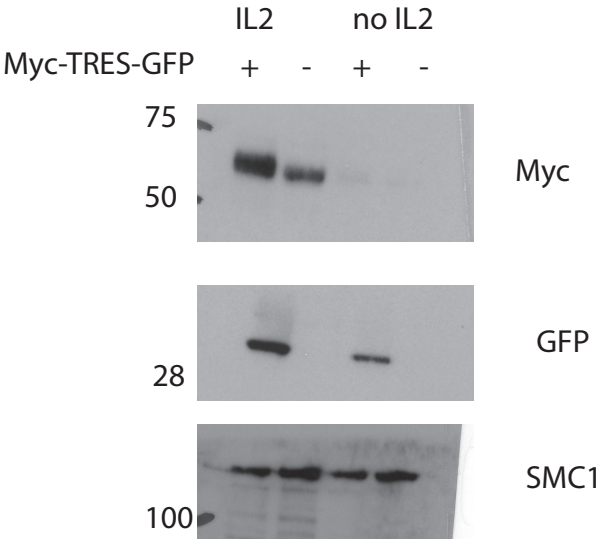

Supplement: Supplementary file 5 [file embj0034-2008-sd5.pdf]

4B

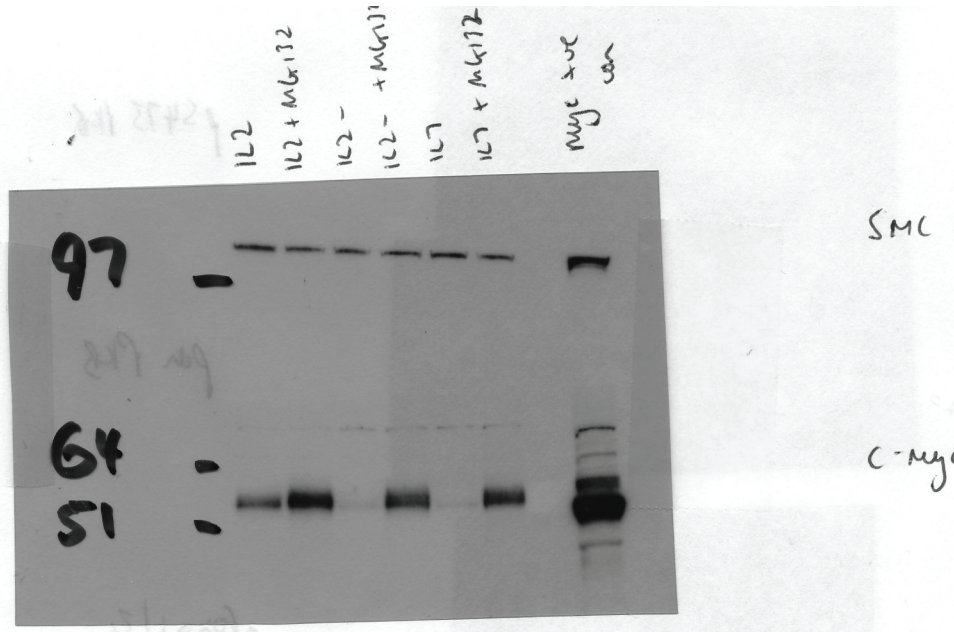

4C

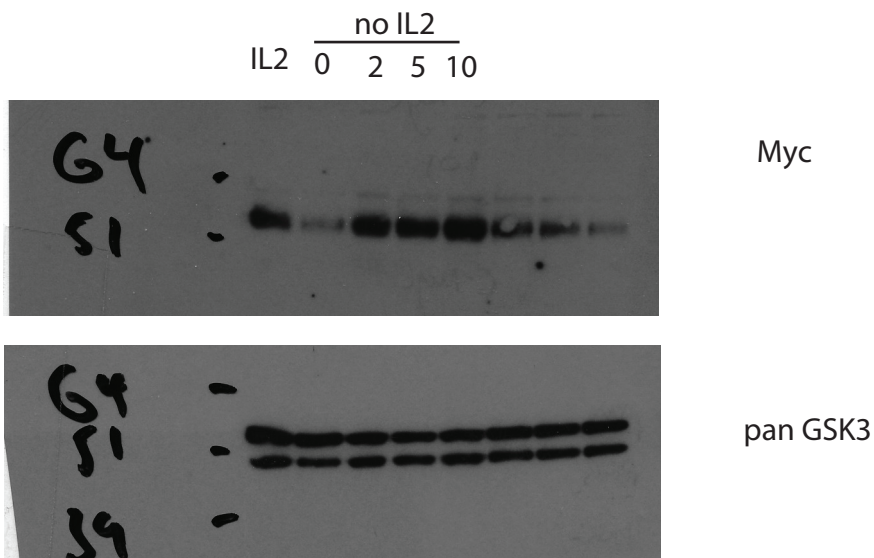

4D

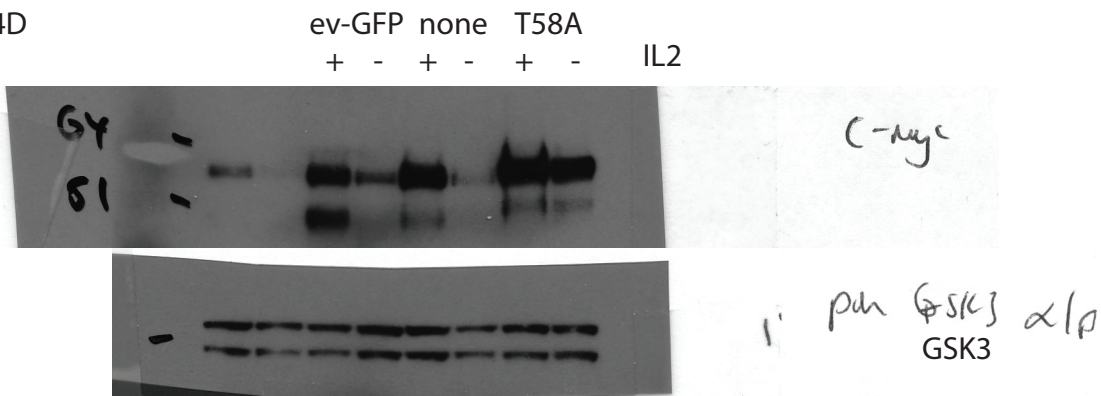

Supplement: Supplementary file 6 [file embj0034-2008-sd6.pdf]

amino acids:      15      30      60      90      120      180      time (min)

amino acids:    +    -    +    -    -    -    -    -

75  
50

cmyc

150  
100

SMc1

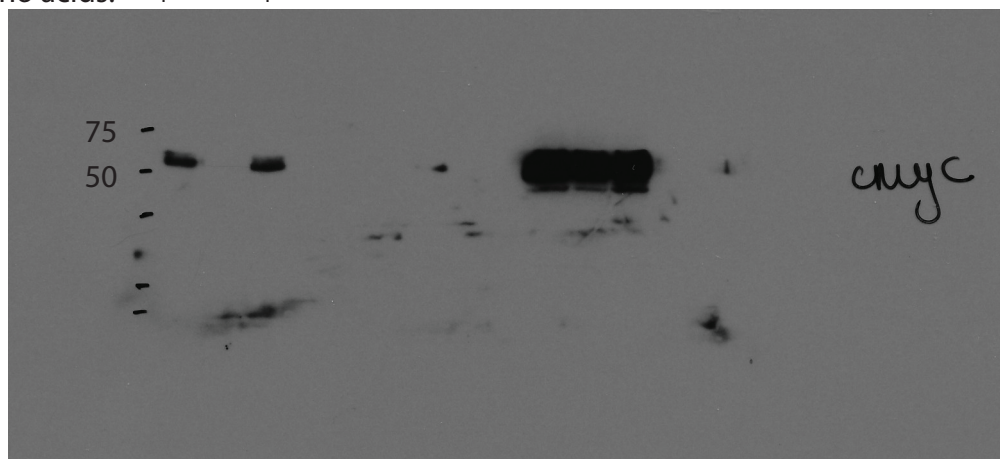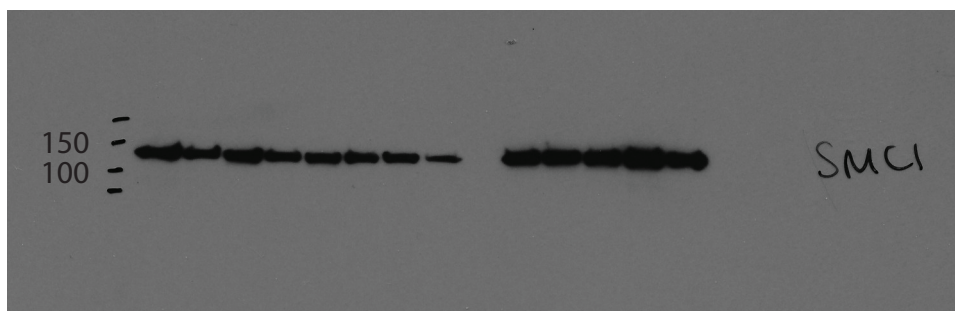

Supplement: Supplementary file 7 [file embj0034-2008-sd7.pdf]
